# Integration of Digestate-Derived Biochar into the Anaerobic Digestion Process through Circular Economic and Environmental Approaches—A Review

**DOI:** 10.3390/ma17143527

**Published:** 2024-07-16

**Authors:** Mohamed Zbair, Lionel Limousy, Méghane Drané, Charlotte Richard, Marine Juge, Quentin Aemig, Eric Trably, Renaud Escudié, Christine Peyrelasse, Simona Bennici

**Affiliations:** 1 Institut de Science des Matériaux de Mulhouse (IS2M), Université de Haute-Alsace, CNRS, IS2M UMR 7361, 68100 Mulhouse, France; mohamed.zbair@uha.fr (M.Z.); meghane.drane@uha.fr (M.D.); simona.bennici@uha.fr (S.B.); 2 Université de Strasbourg, 67000 Strasbourg, France; 3 ENGIE, Lab CRIGEN, 4 Rue Joséphine Baker, 93240 Stains, France; charlotte.richard@engie.com (C.R.); marine.juge@engie.com (M.J.); quentin.aemig@engie.com (Q.A.); 4 INRAE, University of Montpellier, LBE, 102 Av. des Etangs, 11100 Narbonne, France; eric.trably@inrae.fr (E.T.); renaud.escudie@inrae.fr (R.E.); 5 APESA, Pôle Valorisation, 3 Chemin de Sers, 64121 Montardon, France; christine.peyrelasse@apesa.fr

**Keywords:** anaerobic digestion, circular economy, biogas, biochar, digestate, energy

## Abstract

The growing energy consumption and the need for a circular economy have driven considerable interest in the anaerobic digestion (AD) of organic waste, offering potential solutions through biogas and digestate production. AD processes not only have the capability to reduce greenhouse gas emissions but also contribute to the production of renewable methane. This comprehensive review aims to consolidate prior research on AD involving different feedstocks. The principles of AD are explored and discussed, including both chemical and biological pathways and the microorganisms involved at each stage. Additionally, key variables influencing system performance, such as temperature, pH, and C/N ratio are also discussed. Various pretreatment strategies applied to enhance biogas generation from organic waste in AD are also reviewed. Furthermore, this review examines the conversion of generated digestate into biochar through pyrolysis and its utilization to improve AD performance. The addition of biochar has demonstrated its efficacy in enhancing metabolic processes, microorganisms (activity and community), and buffering capacity, facilitating Direct Interspecies Electron Transfer (DIET), and boosting CH_4_ production. Biochar also exhibits the ability to capture undesirable components, including CO_2_, H_2_S, NH_3_, and siloxanes. The integration of digestate-derived biochar into the circular economy framework emerges as a vital role in closing the material flow loop. Additionally, the review discusses the environmental benefits derived from coupling AD with pyrolysis processes, drawing on life cycle assessment investigations. Techno-economic assessment (TEA) studies of the integrated processes are also discussed, with an acknowledgment of the need for further TEA to validate the viability of integrating the biochar industry. Furthermore, this survey examines the techno-economic and environmental impacts of biochar production itself and its potential application in AD for biogas generation, aiming to establish a more cost-effective and sustainable integrated system.

## 1. Introduction

The circular economy model has received considerable attention on the policy agenda, especially in the context of climate change and the decarbonization of the economy. Returning waste to supply chains and meeting the energy demands of local communities and businesses is crucial for rationalizing waste management, increasing resource efficiency, and effectively implementing the circular model. Human demands exert persistent pressure on Earth, and global society continues to grow. Resources are however both critical and limited, and their use must be maximized [1]. Efficient management is essential to promote circularity, combat climate change, and achieve sustainable development [2]. Integrating a circular economy with biotechnology can support local businesses’ growth, while simultaneously preserving the environment from uncontrolled waste disposal [3,4]. An example of this potential is the substantial prospect of reusing approximately 1 million tons of used cooking oil generated in the European Union (EU), often discharged into public sewage systems, elevating wastewater treatment costs [5]. 

The accumulation of municipal and residential waste is increasing as the world population grows, particularly in densely populated areas and in tourist destinations. In addition, agriculture faces challenges, such as significant amounts of agricultural leftovers, livestock residues, and biodegradable waste that are unsuitable for human and animal consumption. The annual global waste generation is estimated between 7 and 9 billion tons, with over 2 billion tons representing municipal solid waste (MSW) [6,7], projected to reach 3.4 billion tons by 2050. 

Since waste management is one of the urgent and crucial issues facing contemporary civilization, efforts are being made to develop technologies to limit waste accumulation in landfills. This includes waste separation in industrialized nations, which makes waste disposal and recycling easier and less expensive. Additionally, organic waste, being predominately biodegradable, can be subjected to anaerobic digestion or incineration. Furthermore, sewage sludge, a byproduct of municipal and industrial wastewater treatment, must be eliminated. Despite the successful reduction of waste that ends up in landfills, the above-mentioned traditional methods can pose various negative environmental implications, including greenhouse gas emissions as well as groundwater, soil, and air contamination. The European Commission, Council, and Parliament have reached a preliminary agreement on the Circular Economy Package of waste-reduction policies. This agreement leads the EU to a good level of waste management sustainability.

Waste-to-gas technology, particularly through anaerobic digestion (AD) in which biodegradable matter is converted into biogas, is recognized as an ecologically sustainable waste management technique, AD can address concerns related to waste management, renewable energy production, sustainable food production, and nutrient recycling in a circular and sustainable manner. In numerous European countries, AD is supplanting emission-intensive waste management alternatives, including landfilling in the agro-industrial sector. Beyond enhancing resource efficiency and reducing CO_2_ emissions, biogas plants bring about a positive economic impact and provide ecologically friendly energy [8]. As part of the development of a circular economy, new approaches could improve the AD sector. 

AD of organic waste is a well-established process that generates biogas, a biofuel used for heat and electricity generation or injection into the natural gas grid after upgrading [9,10]. At the same time, a huge quantity of digestate is produced as residual waste [11]. Digestate contains concentrated organic and inorganic compounds with a high moisture content and agronomic value [12]. Direct use in agriculture may face challenges, such as the quality of the digestate and the limited availability of suitable land near the biogas plant. Given the highly contentious nature of this topic and the extensive research being conducted on the valorization of digestate in agriculture, it is worthwhile to explore alternatives for its valorization. It is worth noting that the unprocessed release of digestate without appropriate treatment can have negative implications for the quality of the surrounding environment [13,14]. Therefore, there is a recognized imperative for comprehensive digestate treatment before its final disposal, even though this process may incur substantial costs [15]. In response to the call to embrace a circular economy in modern societies, substantial efforts have been dedicated to recovering valuable elements from digestate, transforming them into renewable resources.

As a result, using digestate to generate biofuels and value-added products by combining AD with other processes emerges as a promising alternative [11]. Consequently, digestate can be utilized as a feedstock for pyrolysis-based biochar synthesis. Given the possible applications of biochar, it represents a viable and beneficial option for managing this AD by-product [16,17]. Additionally, the circular economy model for digestate handling is attractive for closing the material loop, since digestate could be utilized for biochar synthesis before reintroduction into anaerobic digesters or for various other purposes [18]. The key factors enhancing the efficiency of AD when biochar is added are the physicochemical qualities of the biochar (including its porosity, electrical conductivity, pH, redox properties, etc.) [18]. 

The present review provides an overview of the AD process, critical operational factors, and potential approaches utilized to improve the recovery of organic waste. It also details the valorization of digestate produced by AD, in particular exploring the potential for biochar production from this feedstock and discussing the impacts of biochar on the AD process. As a result, this review aims to elucidate the ability to generate biochar from digestate, examine its impacts on AD when used as additives, and demonstrate its potential benefits for bio-CH_4_ upgrading, thereby illustrating a circular system. The results of this review can guide policy-makers, waste management professionals, and renewable energy developers in implementing more efficient and sustainable waste-to-energy systems. By detailing the potential for integrating AD and biochar production, we provide a roadmap for developing circular waste management solutions that maximize resource recovery and minimize environmental impact. From an academic perspective, this study synthesizes current knowledge in fields such as waste management, renewable energy, and materials science. By linking these generally distinct areas, we aim to identify new research directions and interdisciplinary opportunities in circular bioeconomy systems. This integrated approach can significantly contribute to climate change mitigation, resource conservation, and sustainable development goals by transforming waste management from a linear to a circular model.

Finally, beyond the environmental and economic analysis of combining AD and pyrolysis, the life cycle analysis of the processes under consideration is briefly reviewed.

## 2. Anaerobic Digestion Description

**Figure 1:**
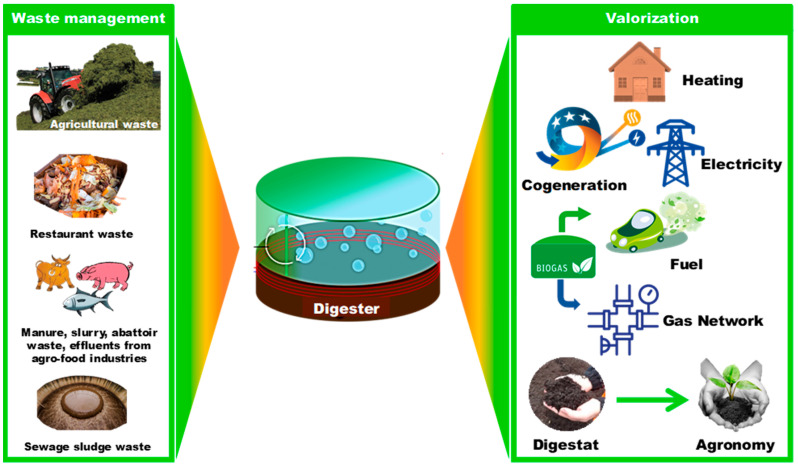
A general description of anaerobic digestion plants.

AD offers the benefit of generating renewable energy, leading to significant growth in the last 20 years. In the absence of oxygen, anaerobic microorganisms transform biodegradable matter mainly into CH_4_, and CO_2_, resulting in a partly stabilized wet organic mixture known as digestate. AD can be conducted as a wet process, with a moisture content exceeding 85%, or as a dry process, with a moisture level of less than 80%. Figure 1 provides a general schematic of the AD process, covering input management to the recovery of AD products and by-products, illustrating the various advantages of this waste treatment approach.

Biogas can be burned on-site to create heat and/or electricity, and if purity standards are met after upgrading, it can be used as a fuel, or injected into the gas grid. The residual digestate consists of a moist solid or liquid suspension of non-biodegradable components, such as non-biodegradable organic waste, microorganisms, microbial residues, and digestive byproducts. The digestate is a partially stabilized wet mixture that may be separated into solid and liquid fractions [7,19]. All organic biodegradable components, such as carbohydrates, proteins, and lipids, can theoretically be employed for AD. 

### 2.1. Mechanisms of AD

**Figure 2:**
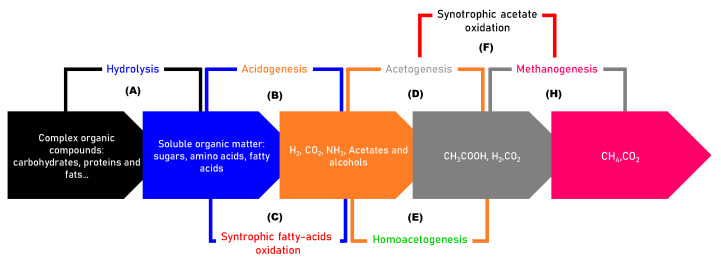
General description of the anaerobic digestion process and pathways.

AD encompasses four biological steps depicted in Figure 2:

- Hydrolysis (stage A): Complex materials, such as proteins, lipids, cellulose, and others, are broken down into simpler substances, including sugars, peptides, glycerol, amino acids, and fatty acids. This transformation is facilitated by exoenzymes produced by anaerobic bacteria [20]. Hydrolysis is a prerequisite for subsequent stages, as it makes the organic material more accessible to microbial degradation.
- Acidogenesis (stage B): Hydrolysis products are converted into volatile fatty acids VFA (e.g., propionate, butyrate.) and other intermediate compounds, such as lactate and alcohol. Some bacteria collaborate with methanogens to further metabolize VFA through syntrophic fatty acid oxidation [7,21].
- Acetogenesis (stage D): The by-products of acidogenesis are transformed into compounds such as acetate and H_2_ by various pathways [22]. Homoacetogenesis is another acetogenesis mechanism in which H_2_ and CO_2_ are used to generate acetate.
- Methanogenesis (stage H): Methanogenic archaea convert acetate and hydrogen into CH_4_ and carbon dioxide CO_2_. There are two main pathways: acetotrophic methanogenenisis, which convert acetate into methane, and hydrogenotrophic methanogens, which utilize hydrogen and carbon dioxide to produce methane. Because of the slow growth rate of methanogens and their sensibility to H_2_ and VFA accumulation, this is one of the most critical steps of AD.

Understanding these steps is essential for optimizing anaerobic digestion and exploring potential synergies with biochar to improve efficiency.

Various types of microorganisms are involved in the different stages of the interdependent conversion process (see Table 1 for examples) [23]. These microorganisms exhibit variable biokinetics and bioenergetics, contributing to an inherent imbalance between substrates and products, often leading to AD instability. In particular, fermentative bacteria, mainly from the *Firmicutes and Bacteroidetes phylum*, lead to the accumulation of VFAs, a major instability factor [24]. These microbial groups, which are very active in AD bioreactors, generate high concentrations of VFAs. However, bacteria that consume VFAs (e.g., syntrophs and acetate-oxidizing syntrophic bacteria) exhibit poor VFA degradation kinetics, only starting to decompose when the H_2_ partial pressure in the bioreactor falls below 10−3 atm [25,26]. Consequently, a collaborative effort between syntrophs and methanogens becomes crucial for the efficient conversion of VFAs to final products (CH_4_ and CO_2_). This symbiotic relationship is essential to ensure AD stability and efficient conversion of organic matter into desired products. Maintaining key operating parameters/factors within the ideal range is essential to ensure the stability and efficiency of the AD process. Table 1 gives examples of the dominant microbial functional groups involved in the anaerobic digestion process, their respective substrates and products, as well as representative examples.

### 2.2. Important Factors Affecting AD

The conversion of organic matter by the AD process into CH_4_ is a complex process that involves a number of distinct degradation pathways. The microorganisms participating in the process may vary for each degradation phase, thereby necessitating different environmental conditions [27].

#### 2.2.1. Feedstock (Substrate)

Many substrates derived from agricultural waste, municipal solid waste (MSW), agro-industrial waste, and energy crops can be used in AD [28]. The physical properties and chemical compositions of the raw material play a crucial role in promoting the biological decomposition necessary for CH_4_ production. Not all substrate constituents—proteins, lipids, and polysaccharides like cellulose, hemicelluloses, and lignin- can be easily decomposed and are therefore biodegradable. As an example, cellulose requires a few weeks to degrade, while lipids, proteins, and hemicelluloses need only a few days. VFAs and alcohol degradation kinetics are much faster (a few hours), whereas lignin is largely resistant [28]. Therefore, selecting suitable feedstocks is crucial considering the quantity of energy generated and waste management. 

Lipids have the largest CH_4_ potential among the major components, but they can hydrolyze into long-chain fatty acids (LCFA) that can inhibit the degradation process, leading to instability and latency issues [29]. Accumulation of LCFA results in damage to bacterial cell walls, disruption of nutritional and metabolic transfers, and process restrictions [30]. The substantial amounts of proteins and lipids present in food wastes produced by the meat industry, food manufacturing industries, and, in some cases, MSW also impact the production of NH_3_ and H_2_S. High NH_3_ concentrations are hazardous for methanogens and can also cause a pH increase [31]. Furthermore, substrates containing high amounts of lipids can lead to additional technical issues, such as clogging of pipes and pumps, foaming, sludge floating, plugging of gas collectors, and problems with substrate and product transport restrictions [32]. However, despite these challenges, lipid-rich raw materials can produce more CH_4_ when associated with other substrates (such as MSW [32], sewage sludge [33], and paper waste [34]).

CH_4_ can also be produced from high-protein-based substrates (i.e., waste from food, fish, and seaweed). The main issue connected to protein decomposition is the generation of a significant NH_3_ concentration, which frequently results in process inhibition or instability. Trying to adjust the pH inside the reactor by introducing acidic iron and acid [35,36] is suggested for overcoming some of the process issues associated with protein-rich raw substrates. In addition, substrates with high contents of carbohydrates, including lignocellulosic biomass, can also be useful for producing CH_4_. Although lignocellulosic biomass (wheat, straw, and grass) has a relatively high theoretical CH_4_ potential, its high lignin content prevents it from being hydrolyzed by microorganisms [37] and makes it resistant to anaerobic digestion. 

The residues from livestock, such as manure or slurry are a frequent source of feedstock for AD. Animal manure is particularly significant and is used as a substrate in the majority of anaerobic digesters in Europe [38]. Using manures to produce CH_4_ in AD reduces the anthropogenic greenhouse gas emissions that would normally be expelled during its storage [39]. The CH_4_ potential of animal manure can widely differ due to various reasons, including the animal’s breed, species, growth phase, diet, and quantity and kind of bedding included in the manure. Manure-derived feedstocks supply crucial nutrients for microbial growth [40] and act as a buffer for the decomposition of low-nitrogen substrates. 

Pretreatment improves the use of raw materials in AD [41]. This approach is recommended for substrates made of extremely resistant matter or matter that is not readily biodegradable. These techniques vary depending on the substrate and approach but generally include thermal, chemical, physical/mechanical, microwave, ultrasonic, and biological methods. Research indicates that thermal pretreatment, covering temperatures from 70 °C to 275 °C, can significantly improve AD efficiency by enhancing feedstock hydrolysis [42,43,44,45]. For example, steam explosion treatment at 210 °C for 10 min significantly increases biogas production from lignocellulosic biomass [46]. However, the variable results from one study to another underline the need for consistent optimization.

The combination of heat and chemicals can reduce feedstock size, increase solubility, and promote the removal of volatile solids, thereby improving AD efficiency [47,48,49]. Acids, alkalis, and additives accelerate the decomposition of specific materials, but their cost-benefit ratio needs to be carefully assessed [50,51,52].

Mechanical methods such as grinding and sonication have shown promise in improving digestibility and CH_4_ production [53,54]. Ultrasound, for example, weakens cell walls, thereby facilitating degradation, albeit with variable effectiveness depending on feedstock characteristics [55]. Microwave irradiation improves access to enzymes, but industrial-scale limitations persist [56].

Enzymes such as cellulases and xylanases have been studied to break down lignocellulosic materials, but their implementation presents difficulties, particularly in terms of cost and efficiency [57,58]. These various pretreatment methods offer opportunities to optimize AD efficiency, but a thorough understanding of their effectiveness, costs, and applicability to various feedstocks is essential for their practical implementation.

#### 2.2.2. Temperature

Operating temperature is an important parameter influencing AD since it determines microbial kinetics and thus the structure of the microbial community present in the digester [27]. Maximum anaerobic digestion rates (gas generation rates, microbial growth rates, and substrate conversion rates) occur at two temperature ranges corresponding to the growth of two specific microbial families: thermophilic (45–60 °C) and mesophilic (25–40 °C) [59]. The temperature regulation can then drive the growth of a specific microbial group. The benefits and drawbacks of using thermophilic or mesophilic microbial populations are listed in Table 2. Both families have been widely utilized to produce biogas from a variety of wastes, and both present various advantages and issues.

#### 2.2.3. pH

Digester efficiency and stability are closely linked to pH. Methane-producing archaea, fermentative bacteria, and hydrolytic bacteria are the three types of microorganisms that are involved in AD. They are sensitive to pH and need a particular pH range to grow. The identification of the optimal pH range for these microorganisms has been investigated in numerous studies. For instance, Boe [62] demonstrated that the optimum pH range for AD is between pH 6.5 and 8.0 and that the pH range between 5.5 and 8.5 is adequate for methanogenic archaea. Moreover, it was reported that fermentative bacteria can operate in a pH range of 4.0 to 8.5 and perform best between pH 5.0 and 6.0 [63]. The reasons why the pH changes are the alkalinity, the presence of VFAs, and the bicarbonate content [64].

Methanogenesis can be negatively impacted by a pH below 6.3 or above 7.8 [65]. The other biological reactions, such as hydrolysis and acidogenesis, are optimized at pH between 5.5 and 6.5 [66]. The pH can be dramatically decreased leading the process to collapse if the intermediates, notably the VFAs generated during acidogenesis, are not degraded. High basicity caused by the increase in ammoniacal nitrogen can also affect AD. In general, extremely acidic or extremely alkaline pH values can be detrimental to acidogenesis [67] and slow down the rate of hydrolysis.

Overloading, inadequate mixing, nutritional deficiencies, temperature change, and loss of microorganisms can all result in high VFA accumulation. If alkalinity is sufficient, organic acids can be neutralized; consequently, buffering reagents may be required. The change in pH can be significantly influenced by the formation of several compounds during biochemical interaction (for example, NH_3_, CO_3_^2−^, and CH_3_COO^−^). As an example, the pH might suddenly increase as a result of the formation of (NH_4_)_2_CO_3_ or the CO_2_ reduction from the digestate liquid [68,69]. The formation of basic cations such as K^+^, Ca^2+^, and Mg^2+^ or the reduction of multivalent anions such as Fe(OH)_3_, or SO_4_^2−^ in the digestate liquid phase can also cause a pH rise. The precipitation of carbonates such as CaCO_3_ or deriving from fatty acids as a result of high organic loading can be crucial in lowering the pH. According to Kovács and coworkers [70], protein-rich substrates such as meat extracts tend to lose some of their ability to function as buffers at high levels of organic loading input, which causes the pH to decrease.

An automated pH controller is frequently used in biogas plants to regulate pH. The controller’s main goal is to balance the pH by supplying the right quantity of a suitable agent. Strong bases (NaOH) or carbonate salts (lNa_2_CO_3_ and NaHCO_3_), as well as acids (HCl), are typically utilized as chemicals to adjust the pH. Decreasing the organic loading rate (OLR) [71], digestate recycling [72], hydraulic retention time variation [73], and co-digestion strategies [74] are some of the other operational factors that can be used to indirectly adjust pH.

#### 2.2.4. Moisture

A critical parameter influencing anaerobic digestion is moisture content, as it plays a vital role in several mechanisms [75]. Moisture contributes to the turgidity of microbial cells, facilitates the transport of microorganisms, products, enzymes, and nutrients, and interacts with complex organic substances during hydrolysis. Additionally, moisture alters the shapes of enzymes and other macromolecules [27] and aids in the dissolution of degradable organic matter. Based on the total solids (TS) content, AD technologies are categorized into three types: low solids or wet digestion (TS < 10%), medium solids or semidry digestion (10% < TS < 20%), and high solids or dry digestion (TS > 20%).

Much research on organic municipal solid waste (OMSW) degradation has used dry AD because of its high solid content [76,77,78]. However, adding water or co-digesting with low-solid wastes, such as manure, can increase the moisture content of OMSW, making it suitable for semi-dry AD [79,80].

According to Lay et al. [81], raising the initial moisture level of mesophilic anaerobic digesters from 90% to 96% enhanced the methanogenic activity in high-solids sludge digestion. In another study [76], digesters with higher starting moisture content produced more methane and had a higher Dissolved Organic Carbon (DOC) removal efficiency in OMSW mesophilic anaerobic digestion. However, increasing the moisture content of OMSW with periodic cycles of leachate drainage and water addition was shown to decrease the methane generation rate in anaerobic digesters [82].

The methanogenesis processes during anaerobic digestion were examined by Hernandez-Berriel et al. [82] at different moisture levels, i.e., 70% and 80%. They discovered that the methanogenic phase began in both cases around day 70. However, bioreactors operating at 70% moisture content led to an increased leachate production, resulting in a higher rate of methane production. Ultimately, the experiment performed at a 70% moisture level produced 83 mLCH_4_/gDM, whereas the one carried out at an 80% moisture level produced 71 mLCH_4_/gDM. They concluded that reactors working at 80% moisture levels produced a lower quantity of volatile solids (VS) than those performing at 70% because water re-additions washed away nutrients and microorganisms [82].

#### 2.2.5. Effect of Carbon to Nitrogen Ratio

Microorganisms require nitrogen as a primary nutrient for their growth [83]. According to Kondusamy and Kalamdhad [83], bacterial utilization of carbon is 25–35 times higher than that of nitrogen. Therefore, a nitrogen-to-carbon ratio of 25–30:1 was suggested for optimal microbial activity in AD [83]. Furthermore, an excess of nitrogen can hinder the process since it leads to the formation of NH_3_. Thus, it can be deduced that C and N are both indispensable for boosting the microbial population and enhancing their growth. Nitrogen in the form of ammonium also contributes to pH buffering, thus playing a key role in AD.

Because of its metabolic byproducts (ammonia/ammonium), nitrogen can pose challenges during AD [84]. While ammonia molecules can penetrate bacterial cells, causing a change in internal pH through conversion to ammonium and, consequently having a negative impact on certain enzyme activities, ammonium ions can directly inhibit the CH_4_-producing enzymes. Temperature and pH affect the percentage of total nitrogen present in the NH_3_ form. An increase in NH_3_ levels inhibits the methanogenic microflora, contributing to the accumulation of VFAs, which, in turn, causes a drop in pH and a corresponding reduction in NH_3_ concentration. A decrease in methane production is thus linked to the presence of NH_3_, the amount of VFAs, and the pH value [84]. AD can be hindered and less robust at thermophilic temperatures than at mesophilic temperatures due to the influence of the temperature on the dissociation equilibrium of NH_3_/NH_4_^+^ [31].

This phenomenon can be mitigated by diluting the solid waste with water to reduce the impact of NH_3_ inhibition [83]. The use of a co-substrate has been shown to affect the methane output at various C/N ratios. Wheat straw, chicken manure, and dairy manure were used as co-substrates in a pH-controlled media. Wang and coworkers [85] demonstrated that a C/N ratio of around 27.2 resulted in the highest methane yield. Karthikeyan et al. [86] found that the maximum CH_4_ output occurred at a C/N ratio of 27 [86]. In fact, providing an adequate amount of carbon can help to avoid excessive ammonia inhibition [87]. Table 3 lists the C/N ratio and CH_4_ yields from co-digestion experiments of OMSW and other organic waste.

#### 2.2.6. Redox Properties Potential Effect

**Figure 3:**
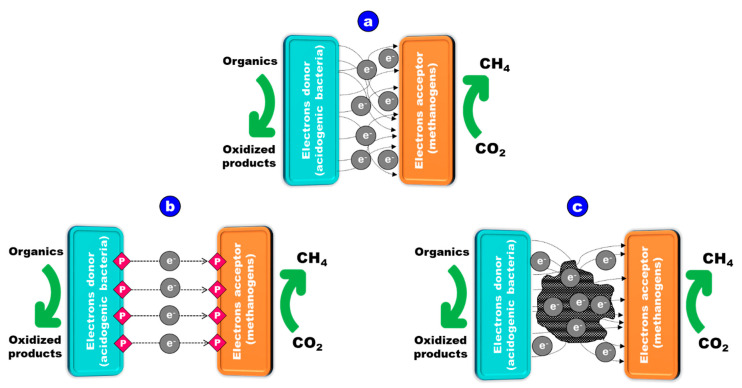
Electron transfer mechanism during AD process. (**a**) Mediated transfer through hydrogen/formate. (**b**) DIET via membrane-bound electron transport proteins (pink). (**c**) DIET via conductive materials.

CH_4_ production results from the interaction between acetogens and methanogens, while electron transfer facilitates the syntrophic interaction between microorganisms. Interspecies Electron Transfer (IET) is therefore essential for maintaining the balance of chemical processes occurring within the anaerobic digester. The successful transformation of organic matter into CH_4_ relies on the transmission of electrons from the electron providers (acidogenic bacteria) to acceptors (methanogens). As shown in Figure 3, there are two ways for an electron to migrate between species [25]: transfer mediated by hydrogen or formate molecules (Figure 3a) and direct transfer of electrons (Figure 3b,c) [25].

##### Mediated Electron Transfer through Hydrogen/Formate

A mediator facilitates the transfer of electrons from the donor to the acceptor. In the syntrophic interaction between acidogenic bacteria and methanogens, hydrogen is an extensively studied mediator that serves as an electron transporter. Notably, formate also contributes to CH_4_ generation by the same pathway as H_2_ [95]. This IET mechanism involves the generation of H_2_ by secondary fermenting bacteria and its use by hydrogenotrophic methanogens [96]. Thermodynamically, the synthesis of H_2_ from VFA or alcohols is only when H_2_ is lower than 10^−4^ atm [97], is possible (i.e., ΔG < 0) [97]. This condition is attained by the consumption of H_2_ by hydrogenotrophic methanogens [98]. The survival of H_2_-producing bacteria and hydrogenotrophic methanogens is therefore dependent on syntrophy [99]. Low H_2_ quantities also limit the rate of CH_4_ production [100]. IET using H_2_ is supposed to be the bottleneck in the synthesis of CH_4_ [101].

##### Direct Interspecies Electron Transfer (DIET)

Methanogens have the capacity to directly receive electrons from donors, a process known as DIET, in addition to the mediated transfer of electrons. The c-type cytochrome receptor on the cell membrane, pili, and conductive materials facilitate DIET between cells [102]. Syntrophic microorganisms, equipped with DIET capacities, are believed to have evolved for interactions with other microbes, evident in their possession of flagella and pili. The establishment of physical interaction with other syntrophic microorganisms depends on these cell appendages [103]. Besides, the presence of membrane cytochromes supports syntrophic bacteria capable of DIET [104].

Conductive materials such as Fe_3_O_4_, Fe_2_O_3_, conductive polymeric materials, and carbon-based nanomaterials [105,106,107] can enhance DIET in exoelectrogenic bacteria and methanogenic archaea. Compared to DIET enriched with conductive materials, conventional AD involves indirect IET via electron carriers like hydrogen or formate, which is less efficient [108]. For effective anaerobic digestion through conductive materials-mediated DIET, two types of microorganisms are essential: CH_4_-forming archaea and electron-donating bacteria.

The bacteria oxidize organic matter, transferring electrons extracellularly to conductive materials [108]. Methanogenic archaea convert CO_2_ to CH_4_ using the electrons received from the electron-donating microorganisms through conductive materials [108]. 

The addition of conductive materials to the AD accelerates the development of methanogens capable of electron transport between various species. The presence of conductive materials enhances the DIET process, establishing an electrical channel between species engaged in DIET [101]. The “lag period”, denoting the time required for bacteria to adapt to the new environment [106], is reduced by 10–75% with the addition of conductive materials to the reactor r [108]. Moreover, the use of conductive materials increased CH_4_ yield and t production rate by 79–300% and 100–178%, respectively [108], owing to faster electron conduction via controlling media [108].

Based on a literature review, it was shown that methanogens in pure cultures require a redox potential of about 350 mV to generate a strongly reducing environment optimal for bacteria function [109]. The term “ORP” stands for Oxidation-Reduction Potential, representing a material’s ability to either accept or donate electrons. In the context of AD, ORP is determined by the presence of both reducing agents (i.e., hydrogen) and oxidizing species (i.e., oxygen or nitrate ions). An ORP over 50 mV indicates an anaerobic environment with free oxygen; between 50 and −50 mV indicates an anaerobic environment; and below −50 mV indicates a reducing environment [110]. Moreover, when ORP is between −50 and −100 mV, the concentration of sulfate-reducing bacteria is larger than that of methanogens. Consequently, sulfate is chosen as a thermodynamically favorable electron acceptor [111,112]. Wang et al. [113] adjusted an anaerobic digester by adding FeCl_3_ (10 mM) to modify its oxidation-reduction potential (ORP). They found that lowering the voltage in the control reactor from 100 mV to −350 mV increased the ORP while adding FeCl_3_ increased it from −350 to −280 mV. Higher ORP levels, beyond −150 mV, led to the production of propionic acid, which had a negative impact on methane synthesis. Wang and colleagues suggested ORP levels below −150 mV to avoid propionic acid accumulation and promote efficient methane production [113]. According to Khanal’s study [114], the decrease in sulfide content resulting from the beneficial effect of oxidation-reduction potential (ORP) typically leads to the inhibition of CH_4_ production. The authors [114] also found that introducing O_2_ to the digester can raise the ORP from −230 to −180 mV, effectively reducing the sulfate concentration to a negligible level. This, in turn, increases the activity of CH_4_-producing bacteria (methanogens), leading to improved CH_4_ generation and enhancing the overall effectiveness of AD.

## 3. Digestate Valorization and Biochar Production in a Circular Economy

### 3.1. Overview of Digestate Valorization

Digestates, characterized by high moisture content, are organic/inorganic mixtures generated as residual waste generated during the AD [12]. Extensive research has demonstrated various potential applications for anaerobic digestate, including its effective use as an organic fertilizer [115] and applications in fertigation, a method allowing simultaneous fertilization and irrigation of crop fields [115]. However, challenges arise due to its substantial water content, resulting in costly transportation [116]. Furthermore, there is a potential for the presence of pathogens, fungal spores, or residual anaerobic micro-organisms from the digestion process, posing environmental pollution and consumer safety concerns. Consequently, it is imperative to subject raw digestate to appropriate treatments to yield a more stable product suitable for agricultural purposes [117]. One effective treatment approach is thermochemical treatment, such as pyrolysis. Pyrolysis can produce biochar, which has the capacity to partially substitute for inorganic fertilizers, thus enhancing soil fertility, mitigating nutrient leaching, and reducing soil erosion [118]. However, it is worth noting that the predominant focus has traditionally been on maximizing biogas production, while the full potential of digestate remains underutilized [119]. Recognizing contemporary society’s commitment to sustainable development, the concept of a circular economy has gained significant traction. Direct disposal or use of some untreated digestate poses a substantial risk to the quality of the surrounding environment [13,14] and contributes to significant greenhouse gas (GHG) emissions [120]. Therefore, stringent treatment measures are imperative prior to the final disposal of digestate [15]. To align with the growing demand for a circular economy in contemporary society, considerable efforts have been directed towards recycling digestate as a valuable renewable resource. This approach not only addresses environmental concerns but also supports the sustainable utilization of resources within a circular framework.

The research focus on “digestate valorization” has significantly intensified in recent years, reflecting its growing importance in sustainable waste management and resource recovery. As the volume of digestate produced through anaerobic digestion processes continues to increase, finding efficient and environmentally friendly methods for its utilization has become a priority. This section provides a comprehensive overview of digestate valorization, examining its composition, potential for resource recovery, and various innovative approaches for its effective use.

The potential utility of digestate varies depending on its elements and their chemical forms. Digestate includes a variety of organic substances (C) and macronutrients (N, P K, Na, and Ca) that may be recoverable. Table S1 (Supplementary Information) provides a non-exhaustive list of the compositions of digestates mentioned in the literature. On the other hand, a viable strategy involves using digestate to produce sustainable fuels and goods with additional value by combining AD and other processes [11]. As a result, digestate can serve as a raw material for the synthesis of biochar through thermochemical processes like pyrolysis. Given the potential applications for biochar, it represents a viable and efficient solution for managing this AD residue [16,17,121]. Additionally, a circular economy approach to digestate management is intriguing for closing the material flow loop, since digestate can be employed to produce biochar, which can then be used in the AD process for other purposes [18].

Analyzing Table S1 reveals significant diversity in digestate compositions. This diversity can justify the varied nature of biochar produced from these digestates. An interesting feature of this diversity is the wide spectrum of carbon content exhibited by different raw materials. For instance, food waste digestate has a notably high carbon content (approx. 42.1%), compared with cow manure digestate, which has a lower carbon content (approximately 39.11%). This wide range of carbon content plays a key role in attributing divergent properties to biochar, including variations in surface area and porosity. In particular, Table S1 highlights differences in nutrient content, encompassing essential elements such as nitrogen (N), phosphorus (P), sulfur (S), and a range of essential metals. These disparities significantly influence the multifaceted utility of biochar, from its nutrient retention capacity to its potential as an effective soil amendment in agricultural ecosystems. Discrepancies extend even to elemental composition, with variations in the presence of elements such as sodium (Na), magnesium (Mg), potassium (K), calcium (Ca), iron (Fe), silicon (Si), and aluminum (Al). These variations noticeably influence the ash content and inherent mineral composition of the final biochar product. In summary, this diversity in biochar characteristics makes it a versatile material with potential applications in agriculture, environmental remediation, and other fields. The specific properties of biochar can be tailored to suit different purposes based on the feedstock used in its production, thereby underlining its relevance within the framework of AD. This section highlights the potential integration of AD with the thermochemical process is highlighted in this section, emphasizing the benefits of a circular approach that combines multiple operations [122]. In such an integrated system, digestate can either be gasified or pyrolyzed to produce biochar, syngas, and bio-oil, which can then be decomposed anaerobically [123,124,125,126,127]. The biochar generated can be reintroduced into AD to enhance its efficiency and increase CH_4_ [128,129,130,131]. The physicochemical features of biochar, including porosity, pH, electrical conductivity, cation exchange capacity, functional groups, and redox properties, play a crucial role in elevating microbial performance and enhancing the AD process [18]. The introduction of biochar to AD can improve alkalinity and the pH of the media [128], and the microbial community can face lower acid stress and ammonia inhibition. The large specific surface area (SSA) and porous nature of biochar can favor the colonization of bacteria and archaea, thereby enhancing AD performance [132]. Biochar, with numerous surface functional groups and good electrical conductivity, might increase CH_4_ production through direct or indirect electron transfer mechanisms between anaerobic microorganisms [133,134]. Additionally, undesired components such as siloxanes, CO_2_, H_2_S, and NH_3_ can be effectively removed from biogas (purification) using biochar as adsorbents [135,136,137,138]. This approach, utilizing renewable raw materials, not only supports the development of a low-carbon economy but also combating climate change by producing non-fossil biofuels (biogas). This aligns with the concept of a circular economy, representing a sustainable development approach that contrasts the linear production system. 

Given the potential application of biochar in AD, it becomes crucial to focus on the circular integration of biogas production and thermochemical processes. As a result, the importance of this overview lies in delineating the potentiality of producing biochar from digestate, elucidating its effects on AD when used as an additive, and highlighting biochar’s potential application for biogas purification. The ultimate aim is to propose a circular system. 

### 3.2. Biochar Production from Digestate

**Figure 4:**
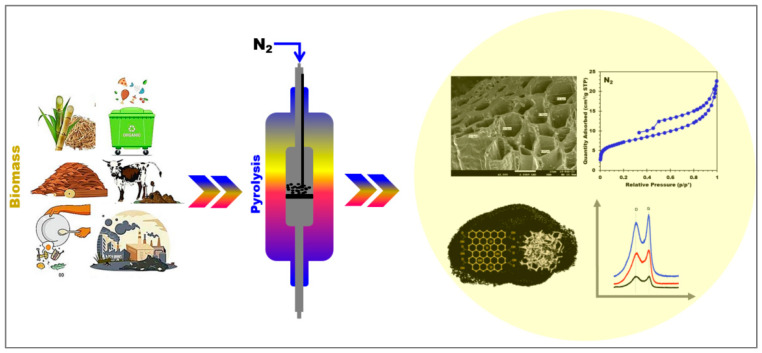
Schematic representation of biomass conversion to biochar and its characterization.

In an oxygen-free environment, pyrolysis is a thermochemical process that transforms organic materials (waste biomass) into syngas, bio-oil, and biochar. The quality and characteristics of biochar derived from digestate depend on the settings of the thermochemical process and the origin of the digestate (Figure 4). Table 4 provides properties of numerous biochars prepared from digestate. 

Many research studies have shown that the physicochemical properties of biochar are strongly influenced by the origin of feedstocks, fabrication methods, and associated process parameters (e.g., carrier gas, temperature, heating rate, and residence time) [139]. Biochar properties can be tailored for specific applications based on selected synthesis parameters. Features like porosity, specific surface area (SSA), cation exchange capacity (CEC), electrical conductivity (EC), redox characteristics, pH, and surface functional groups are crucial to AD [134,140]. 

The high SSA of biochar is associated with its porous (micro, meso, and macro) structure. In comparison to granular activated carbon (GAC) (1 µm), the macropore size of biochar was taller and broader (1–40 µm)) [141]. Biochar would be preferable to GAC as an additive in AD because of its larger macroporous size, promoting the development of microbial communities [134]. In addition, biochar typically maintains a pH above 7, aiding in balancing the inherent acidic AD environment and enhancing the richness of microbial populations [142,143].

Biochar is composed of the following major elements such as C, O, and H, as along with heteroatoms like N, P, and S, as well as metal elements like K, Na, Mg, Ca, and so on [144,145]. Ratios like H: C, O: C, and N: C determine the quantity and properties of surface functional groups containing O and N atoms [146,147]. The EC of biochar (0.002–23.8 dS/m) often exceeds that of GAC (3 ± 0.327 dS/m), a crucial factor in the growing use of biochar as an AD additive [148,149]. In general, the adjustable physicochemical properties of biochar make it an attractive material for enhancing AD effectiveness as a pH buffer, adsorbent, catalytic support, microbial habitat, and electron carrier [140,146,150]. Therefore, understanding the physicochemical properties of biochar and their impact on AD is essential for designing biochar with desirable properties for AD applications.

**Table 4:** Characteristics of biochars from various digestate.

Digestate Source	Transformation Protocol	C(%)	H(%)	N(%)	S(%)	O(%)	SSA (m^2^/g) of Biochar	Application	Ref.
Food waste	550 °C	N.R	N.R	N.R	N.R	N.R	N.R	Soil amendment	[151]
Food waste	300−700 °C	36.7–45.4	0.9–4.4	1.9–5.36	0.44–0.63	0.3–9.14	0.66–26.95	Without application	[152]
Food waste	300−700 °C, 240 min	34–45.4	N.R	1.9–5.36	N.R	N.R	N.R	Soil amendment	[153]
Food waste	400−800 °C, 30 min	N.R	N.R	N.R	N.R	N.R	51.15–57.43	Without application	[154]
Food waste	500 ± 50 °C, 45 min(pilot scale)	11.08	0.8	0.82	0.15	10.05	51.15–57.43		
Municipal waste	400–450 °C	17.1	0.8	0.9	N.R	1.5	N.R	Soil amendment	[155]
Municipal waste	600–650 °C	18.5	0.5	0.6	N.R	0	N.R		
Municipal biowaste	540 °C, 60 min	72.7	2.2	7.2	N.R	N.R	0.51	Without application	[156]
Swine manure	500 °C, 60 min	50.3	1.1	1.4	0.7	7	N.R	Adsorption	[157]
Swine manure	500 °C, 60 min, steam activation 800 °C, 30−60 min	31.1–39.6	0.5–1	0.2–0.3	N.R	0.2−7.6	411–432		
Swine manure	800 °C, 30 min	N.R	N.R	N.R	N.R	N.R	101.9	Without application	[158]
Swine manure	550 °C, 120 min	N.R	N.R	N.R	N.R	N.R	17.07	Adsorption	[159]
Swine manure	550 °C, 120 min HCl, NH_3_, Mn modification	N.R	N.R	N.R	N.R	N.R	186.5–207		
Swine manure	550 °C, 120 min	53.02	8.88	4.3	N.R	N.R	N.R	AD	[128]
Corn	600 °C	81.9–84.6	N.R	N.R	N.R	N.R	N.R	Without application	[160]
Corn	400, 600 °C	41.3–43.8	0.86–1.21	1.58–1.91	N.R	3.8–3.87	N.R	Without application	[161]
Cow manure/food wastes	800 °C, 240 min	58.99	N.R	1.25	N.R	N.R	N.R	Without application	[162]
Corn silage, cow manure	400–800 °C, 30 min	76.2–88.3	2.8–5.19	2.1–3.69	0.72–0.77	5.9–14.1	161.6 (800 °C)	Without application	[163]
Corn silage, manure, and vegetable waste	500 °C, 180 min	55.83	2.03	1.46	N.R	N.R	N.R	Soil amendment	[164]
Pig manure	800 °C, 30 min	21.08	0.45	1.09	2.33	10.23	110	Without application	[165]
Pig manure	550 °C, 120 min, MnO_2_ impregnation	76.59	N.R	N.R	N.R	5.24	16.09	Catalysis	[166]
Rice straw	500 °C, 120 min	48.2	2.6	1.71	0.15	N.R	37.53	Adsorption	[167]
Rice straw	500 °C, 120 min, CuCl_2_ H_2_O with NaBH_4_	38.68	2.03	1.29	0.3	N.R	135.35		
Agroindustrial residues, herbaceous biomass	500 °C, 180 min	52.1	N.R	1.38	N.R	N.R	N.R	Soil amendment	[168]
Animal sewage, cow manure, maize, triticale silages, cereal bran	600 °C, 10 min	57.3	2	1.4	0.07	7.2	88	Soil amendment	[169]
Cattle manure, maize silage	350, 550 °C, 60 mint	60.7–65.9	N.R	2.2–2.6	0.3–0.5	N.R	N.R	Without application	[170]
Cattle manure, pig manure maize silage	300−600 °C, 30 min	56.3–57.2	N.R	2.6–2.9	N.R	N.R	N.R	Soil amendment	[171]
Dairy cattle slurry, silage	400−600 °C, 60 min	42.9–50.6	1.55–2.3	1.88–2.3	N.R	45.4–52.8	11.3–15.3	Adsorption	[172]
Dairy cattle slurry, silage	600 °C, 60 min, urea modification	53.1–53.5	1.1–1.78	2.4–8.99	N.R	35.9–43.3	6.8–15.1		
Groats, olive oil cake, silage of triticale, chicken manure	600 °C, 10 min	62.2	2	1.5	0.04	11.4	49	Soil amendment	[169]
Herbaceous biomass, agro-industrial residues	500 °C, 60 min	64.34	2.68	1.78	0.22	6.38	23.1	Without application	[121]
Maize	550 °C	N.R	N.R	N.R	N.R	N.R	27.5	Adsorption	[173]
Sewage sludge	550 °C	N.R	N.R	N.R	N.R	N.R	N.R	Without application	[174]
Sewage sludge	300−550 °C, 15 min	N.R	N.R	N.R	N.R	N.R	N.R	AD	[175]
Corn straw	700 °C, 60 min	N.R	N.R	N.R	N.R	N.R	335		[176]
Organic fraction of municipal solid waste (OFMSW)	300–700 °C, 120 min	N.R	N.R	N.R	N.R	N.R	N.R		[177]

Table 4 provides a comprehensive overview of the application of biochar produced from various digestate sources in different fields, with a particular focus on soil amendment. It is noteworthy that there is a lack of studies exploring the application of these biochars derived from digestates of AD. Biochar production processes from different digestate sources exhibit substantial variations in temperature, duration, and transformation protocols, resulting in diverse biochar compositions and characteristics. For instance, in the context of food waste AD, biochar produced at 550 °C lacks reported values for carbon (C), hydrogen (H), nitrogen (N), and sulfur (S) content. Varying production conditions for food waste-derived biochar results in varying C, H, N, S, and oxygen (O) content, reflecting the sensitivity of biochar properties to production conditions. However, specific applications from these types of biochars have not been reported. Municipal waste biochar, generated at different temperatures, exhibits variations in C, H, N, and O content. While one study suggests its application in soil amendment, further research is required to explore its full potential. In contrast, swine manure-derived biochar, produced under various conditions, exhibits a wide range of compositional variations in, H, N, S, and O content, finding applications in adsorption, catalysis, or remaining unapplied. Biochar derived from corn, cattle manure/food waste, and various agricultural feedstocks presents similar compositional variations influenced by production conditions; yet specific applications are often unspecified. Interestingly, dairy cattle slurry and silage-derived biochar, while displaying compositional diversity, have primarily been applied in adsorption processes. Furthermore, the SSA of biochars varies significantly among different sources and production conditions. This parameter can impact microbial attachment, growth, and enrichment, particularly in soil amendment applications.

It is essential to emphasize that while these studies offer valuable information on the production of biochar from digestates and their potential applications, comprehensive characterization is often lacking. In many cases, essential information, such as the influence of these biochars on AD processes, underlying mechanisms, and their effects on biogas production, is frequently absent. This lack of detailed data on biochar properties poses a significant challenge in accurately determining biochar characteristics that influence the AD process. This research gap has far-reaching implications. To comprehensively understand how biochar influences AD, it is imperative to undertake comprehensive characterizations of the biochar materials used. Without this essential information, establishing clear links between biochar properties and AD performance becomes a challenging task. Future studies in this field should prioritize in-depth biochar characterization, in order to elucidate the mechanisms involved and the potential benefits of deploying digestate-derived biochar in anaerobic digestion processes. This concerted effort will enable researchers to fully exploit the potential of biochar as an additive to AD, improving biogas production and optimizing waste management. In addition, it should be noted that substantial research has been carried out on the use of biochar derived from various biomass sources in the AD process. These studies will be discussed in the following section, highlighting their specific properties and contributing to the understanding of the impact of digestate-derived biochar on AD processes.

## 4. Biochar in AD: Investigating Properties and Outcomes

In view of their potential to enhance CH_4_ production, conductive carbon materials, such as granular activated carbon (GAC) and biochar, are gaining attention as promising additives [178,179]. Biochar provides a range of potentially valuable benefits compared to other additives, as it can be customized for specific applications by adjusting the feedstock, pyrolysis conditions, and activation mechanism [180]. As a result, numerous investigations have supported the potential for CH_4_ production through the incorporation of biochar [140,181,182,183,184,185,186,187,188,189,190].

The addition of biochar in AD has demonstrated improvement in the quality of digestate, particularly in terms of nutrient preservation, increased C/N ratio, and reduced nutrient leaching [191]. Moreover, once biochar has completed its role in AD, it is not required to be separated from the digestate.

While the influence of biochar supplementation in AD is the subject of a growing number of investigations, several unresolved issues persist. For instance, it would be useful to better understand how the buffering capacity of the AD system can be enhanced, how inhibitory phenomena can be mitigated, how syntrophic metabolic functions can be enhanced, and how biogas can be purified. Relationships between biochar properties and their impact on AD are outlined, and the environmental and economic impacts of biochar application are reported.

Table S2 (Supplementary information) provides a concise overview of the application of biochar in AD, highlighting its multifaceted benefits. These advantages include enhanced methane production rates, shortened lag phases, improved buffer capacity, facilitated electron transfer, and the targeted influence on microbial populations. The effectiveness of biochar is contingent upon its source and the temperature at its production, emphasizing the importance of tailored approaches in AD systems. Moreover, the capacity of biochar to stabilize pH s and mitigate VFA accumulation is particularly remarkable. These insights can be invaluable in the controlled biochar production from digestate, with desired properties for AD processes. Examining the specific properties of biochar from Table S2 reveals that two factors consistently play a significant role:

- Buffering Capacity: Several studies highlight biochar’s ability to stabilize and maintain pH levels within AD systems as a critical factor. This buffering capacity is crucial in preventing rapid pH drops resulting from VFA accumulation, thereby maintaining the activity of microorganisms responsible for methane production. Biochars with higher alkaline content, including alkali and alkaline-earth metals such as sodium, potassium, calcium, and magnesium, tend to exhibit superior buffering capacity, creating an environment conducive to efficient AD.
- Specific Surface Area: Another common factor is the specific surface area of biochar. Biochars with higher SSA provide ample attachment sites for microorganisms, promoting biofilm formation and facilitating the colonization and growth of microorganisms essential for AD. Additionally, a higher specific surface area may enable the adsorption of VFA, influencing the overall efficiency of the AD process.

While these two properties, buffering capacity, and specific surface area, consistently stand out, it is important to recognize that the effectiveness of biochar in AD can depend on various other factors, including feedstock source, pyrolysis temperature, and biochar-to-substrate ratios. Therefore, a tailored approach that considers these factors is crucial to fully harness the potential of biochar in enhancing AD processes.

### 4.1. Effect of Biochar pH on AD

pH v is the crucial indicator of AD efficiency, and a decrease in pH has a major negative impact on microbial performance [140]. This decline is typically attributed to VFAs generated as intermediates during t AD [192]. Syntrophic acetogens and methanogens, which transform VFAs into CH_4_ and CO_2_, usually counterbalance this impact [193]. However, under conditions of high organic loading rate, particularly with easily degradable wastes, the accumulation of VFAs can cause a pH reduction and even AD failure [193,194]. The buffer capacity of digestates, which is crucial for neutralizing VFAs, is often considered a rate-limiting step [195,196,197]. The alkalinity in AD, primarily in the form of CaCO_3_ and CO_2_, determines the buffer capacity [192,198]. 

For many reasons, the addition of biochar as a buffer is considered to be an attractive method, given its economical and sustainably sustainable production. The pH of the biochar has a significant impact on the conductivity of the AD media and microbial interactions [199]. Due to the ash content and volatilization of the acidic functionalities, the pH of biochar, typically alkaline, rises when produced at higher pyrolysis temperatures [147,199]. The alkalinity in the AD media was observed to increase with the addition of biochar, resulting in a pH greater than 6, which greatly enhanced microbial activity and CH_4_ production [200]. The rapid VFA formation during AD results in a low pH and certain functional groups present in biochar, such as amines, have the ability to adsorb H^+^ ions and acquire electrons, potentially mitigating the drastic drop in pH. 

The ash derived from biochar contains inorganic components, including Ca, K, Mg, Na, Al, Fe, Si, and S. The alkalinity is mainly attributed to alkali metals and alkaline earth metals. The alkalinity of biochar resulting from ash fraction may significantly increase the buffer capacity and counteract VFA inhibition. In a study conducted by Jang et al. [201], a comprehensive investigation was carried out to explore the effects of biochar derived from dairy manure (DM), on the dry AD process at different temperatures (20 °C, 35 °C, and 55 °C), temperatures. Lower VFA concentrations and a higher CH_4_ production were observed for all temperature scenarios. They suggested that biochar with high nutrient content (9.1% Ca, 3.6% Mg, 1.3% N, and 0.14% P) and potential alkalinity could contribute to increased CH_4_ production. Wang et al. [196] investigated the impact of biochar derived from vermicompost on the ability to buffer high organic loads, such as chicken manure and kitchen waste, in tAD. The study demonstrated the presence of ash, basic functionalities, and the strong buffer ability of this biochar on various VFAs (700–3800 mg/L). 

Quintana-Najera et al. [126] compared the effects of biochars (BC) and hydrochars (HC) on AD in terms of VFA accumulation, pH and CH_4_ production. Biochars, including biochars produced from Oak Wood (OW-BC) and Water Hyacinth (WH-BC), (were more effective in preventing VFA accumulation, with values below 50% of the control, while HC led to more VFA accumulation. This is consistent with previous studies [202,203]. The pH of the systems was also influenced by the type of additives, with higher alkali BCs leading to an initial alkaline pH, while HCs and the control had values closer to neutral pH. The digester, when using biochar produced from *Fucus Serratus* (FS-BC), had an abnormally high pH (~9), potentially impeding VFA consumption and methanogenic activity. At the end of the experiments, digesters supplemented with FS-BC and HCs showed the largest pH changes, with HC reaching detrimental pH levels for methanogens (5.3–6.4). The final pH values for all other BC systems stayed within the ideal range, unlike the HC systems. Due to these pH variations, it is not possible to attribute a buffering effect to either BCs or HCs, suggesting that BCs were more effective than HCs in improving AD, reducing VFA accumulation, and maintaining suitable pH conditions for methanogenesis. Further research could focus on optimizing conditions for different digesters and investigating the effects of different BC and HC production methods on AD performance.

The impact of biochar derived from walnut shells on thermophilic and mesophilic environments AD of food waste was investigated by Linville et al. [188]. This biochar increased stability by raising the alkalinity (pH > 8) from 2800 mg/L to 4800–6800 mg/L CaCO_3_. Similarly, in the primary sludge AD carried out by Wei et al. [143], the presence of biochar derived from corn stover, rich in alkaline-earth metals, demonstrated efficient solids removal and significant CH_4_ production. The increase in alkalinity (3500–4700 mg/L CaCO_3_) resulting from the addition of biochar was associated with an enhancement buffering capability. Ambaye et al. [175] found that sewage sludge biochar supplementation in an AD reactor increased VFA degradation and CH_4_ production. Maintaining the ability of biochar to act as a buffer and determining its inorganics concentration (ash content, alkali, and alkaline earth metals) should be a priority. Since a large dose of inorganics may be detrimental to AD, it is critical to optimize the quantity of biochar required [128,143,175,188].

### 4.2. Effect of Biochar on Electron Transfer Mechanism: Role of Redox Properties

The redox characteristics of biochar emerge as a critical factor in the AD process, with researchers underlining their importance in facilitating methane production and conversion. This insight contributes to a more comprehensive understanding of the role of biochar in enhancing and optimizing AD processes [184].

According to Chacón et al. [204], the redox characteristics of biochar are influenced by its surface functional groups, the existence of free radicals, and the presence of metals and metal oxides. Quinoids (C=O fragments) are identified as the essential functional groups responsible for their electron-accepting capacity (EAC), while phenolic (C-OH) groups contribute to the electron-donating capacity (EDC) [205]. Overall, these groups control the biochar’s electron exchange capacity (EEC; EEC = EDC + EAC). Strengthening the surface functional groups of biochar through the oxidation method is feasible [144,206], but it must be powerful enough to introduce new functionalities without converting them into redox-inactive COOH functionalities or decomposing them into CO_2_ [204]. Aryl radicals (carbon-centered) and semi-quinoid radicals (an intermediate between the phenolic C-OH and quinoid C=O groups) are two types of free radicals that influence the biochar’s redox propensity [207]. Redox-active metals like Fe and Mn oxides, frequently found in the raw material, exist in a variety of oxidation states and can act as electron donors/acceptors concerning the inorganic constituents of biochar [208]. Biochar, similar to granular activated carbon, has been found to facilitate DIET in AD [209]. 

Several investigations have demonstrated that the incorporation of conductive materials, such as granular activated carbon (GAC), into co-cultures of electron-donor bacteria and electron-acceptor methanogenic archaea [210,211] enhanced EIT for ammonia synthesis. It is established by various researchers [210,212,213] that DEIT for methanogenesis is not mediated by diffusive electron transporters (i.e., H_2_ or HCO_2_^−^), but by electrons directly transmitted to methanogenic archaea through conductive materials. It is suggested that conductive materials serve as electrical conductors, thereby facilitating EIT. Notably, most DIET research with conductive materials has shown shorter latency periods for CH_4_ production, higher CH_4_ production rates, high CH_4_ yields, and resistance to inhibitions. These investigations indicate that conductive materials can significantly enhance AD.

The occurrence of DIET has been observed in a thermophilic co-digestion of waste-activated sludge and food waste, attributed to the conductivity and the redox properties of biochar; this phenomenon enhanced co-digestion performance by promoting syntrophic methanogenesis [214]. 

In a reactor supplemented with biochar during the AD of synthetic wastewater, Wang et al. [215] demonstrated an enrichment of the microbial community with potential DIET-partners such as *Geobacter* and *Bacteroidetes*, along with archaea such as *Methanosaeta* and *Methanosarcina*. They proposed that the biochar derived from rice straw might improve COD degradation and a CH_4_ yield by promoting DIET between electrogenic bacteria and archaea, thereby improving the granular sludge’s electron transmission ability. Furthermore, another study by Wang et al. [195] revealed that adding various biochar concentrations to dewatered activated sludge and food waste during the mesophilic AD boosted the CH_4_ production rate and decreased the lag phase [195]. The authors suggested that the buffering capacity of biochar prevented the pH drop caused by the formation of VFAs and the DIET enhancement.

On the other hand, electrical conductivity (EC) plays a significant role in governing syntrophic activities carried out by microorganisms [186,216]. Martins and coworkers demonstrated that the EC of biochar was found to be low, probably influenced by the composition and metabolism of bacterial colonies compared to the EC of the digestate. [217]. despite its significantly low biochar EC, its capacity to promote DIET is comparable to that of granulated activated carbon [108,209]. According to Barua and Dhar [218], microbial community species obtained from AD exhibit a substantial EC of 0.2–36.7 µS/cm due to DIET. 

The importance of the EC of biochar produced from sawdust (SD-BC) and sewage sludge (SS-BC) in the syntrophic oxidation of VFA was assessed by Wang et al. [184]. Since SD-BC contains some redox-active functionalities, it was observed to enhance microbial activity and the decomposition of VFAs through DIET. In addition, the research highlighted an interesting observation regarding the EC of SD-BC and SS-BC, which showed similar values of 0.11 μS/cm and 0.09 μS/cm, respectively. Despite this similarity in EC, a substantial distinction emerged in terms of VFA accumulation between SD-BC and SS-BC. Intriguingly, this divergence suggests that EC alone does not serve as a decisive factor in mitigating VFA accumulation. However, a key contrast emerged in the comparison of SSA, with SD-BC exhibiting an SSA almost 100 times greater than SS-BC, with values of 248.6 m^2^/g and 2.6 m^2^/g, respectively. This significant difference in SSA offers significant advantages, particularly in terms of facilitating microbial attachment, growth, and enrichment [184]. Batch studies in methanogenesis-inhibited systems were conducted to verify the presence of propionate or butyrate in order to validate the biochar’s electron-accepting capacity (EAC) in the syntrophic activity and improvement of acetate formation. The relevance of biochar’s physical qualities and electrical properties in the CH_4_ formation in AD was established by a control test with a non-conductive material [217]. 

It has been observed that the EC of the digesting medium increases in the presence of biochar [215,219]. Despite the fluctuation of the EC of biochar based on the metabolism and species compositions of bacterial communities, the EC of the digesting medium appears to be unrelated to this value [217]. Even though biochar’s EC was nearly a thousand times lower than that of GAC, its capacity to promote DIET seemed to be equivalent to that of GAC [108,149]. According to Martins et al. [217], conductive materials may play an identical role as humic compounds in DIET by serving as electron shuttles capable of both accepting and donating electrons. In AD systems, having another control with a non-conductive material may be critical to determine whether the stimulatory effects of biochar on CH_4_ production may be more broadly associated with the SSA and porosity of biochar than with its EC [217]. However, this finding is clear from the results of the study by Cruz Viggi et al. [220], where two controls were set up, one without biochar and the other with silica sand (non-conductive), for food waste AD. The authors revealed faster VFA decomposition and CH_4_ production in the biochar-modified reactors than in the two control reactors, confirming the major impact of the EC of biochar.

Based on the aforementioned investigations, research on microbial processes of methanogenesis has shown the importance of IET and DIET in CH_4_. Conductive materials, such as biochar, have been shown to enhance electron transfer and methanogenic activity. The EC, redox properties, and surface functional groups of biochars influence their ability to promote electron transfer. EC and redox properties in biochar are primarily influenced by the biochar’s composition and the specific functional groups present on its surface. EC is related to the presence of conductive materials, such as graphitic carbon structures or conductive minerals in biochar. These conductive elements enable the flow of electrons within the biochar matrix. In the context of AD, EC is responsible for providing a pathway for electron transfer between microorganisms involved in the AD process.

Regarding redox properties, biochar’s ability to undergo reduction-oxidation reactions is linked to its surface chemistry. The redox-active sites on the biochar surface, often associated with functional groups like quinones and metal oxides, participate in electron exchange. During AD, these redox-active sites can facilitate the transfer of electrons between microorganisms by acting as intermediaries in redox reactions. This electron shuttle mechanism is critical for ensuring that electrons are readily available for microbial processes, ultimately improving the efficiency of methane production. In summary, the EC of biochar is a result of conductive components within its structure, enabling efficient electron transfer. On the other hand, redox properties are tied to the presence of redox-active sites on the biochar surface, which play a pivotal role in mediating electron exchange between microorganisms during anaerobic digestion. These properties collectively contribute to biochar’s ability to enhance the performance of AD processes. In addition, these studies demonstrate that the addition of biochar increases CH_4_ production, and COD removal, and selectively enriches potential DIET partners in microbial communities. The EC of the biochar plays an essential role in these processes, but its SSA and porosity may also contribute to its effects on methanogenesis.

### 4.3. Effect of Biochar Textural Properties on AD

The porosity of biochar is considered an important factor in the development of the biofilm responsible for AD. Porosity is characterized by pore volume (cm^3^/g) and pore size distribution (<2 nm, 2–50 nm, and >50 nm) [221]. Porosity can induce diverse effects of biochar in AD. Pores smaller than 2 nm and in the 2–50 nm range are crucial for adsorbing small molecules, while pores higher than 50 nm provide a suitable environment for microorganism growth [222,223]. Yin et al. [224] enlightened that higher pyrolysis temperatures of result in larger SSA and porosity. Trigo et al. [225] observed an increase in SSA for various hardwood biochars with an increase in pyrolysis temperature within the 350–700 °C range. In addition, Chen et al. [209] reported improved porous structure in sewage sludge biochar when pyrolyzed between 500 °C and 900 °C. The macrostructure of biochar can provide microhabitats for the attachment and growth of microorganisms [186,216], knowing that the typical sizes of bacteria and archaea involved in AD fall within the range of 0.3–13 µm [186,216]. Moreover, biofilms promoted by biochar porosity can serve as a barrier for the selective enrichment of microorganisms actively involved in AD during stressed conditions, such as an acidification phase [226]. In AD supplemented with biochar, several microorganism species were preferentially enhanced. The separation of the digesting medium into different fractions allowed for the investigation of the spatial distribution of bacteria and archaea [226,227]. Lü et al. [228] described the spatial distribution of methanogens in the biochar pores based on their distinctive morphology and size. Larger methanogens like *Methanobacterium* (1.2–12 µm) face limitations in penetrating biochar pores while smaller *Methanosaeta* (0.8–7 µm) is capable of being dispersed throughout both the external and internal biochar pores [228,229]. This widespread dispersion of microbial populations in biochar pores may enhance CH_4_ produced during AD [186,216]. Indeed, when microbial populations spread widely in the tiny spaces (pores) of biochar, they can potentially increase CH4 production during the AD process.

### 4.4. Effect of Biochar on the Inhibitor Sorption

The presence of inhibitors is frequently cited as a main cause of reduced CH_4_ production and the instability of AD. If a chemical causes a negative change in the bacterial community or stops bacterial development, it is referred to as an “inhibitor” [230]. Direct inhibitors, such as metals and certain organic chemicals like sodium, potassium, copper, pesticides, and others, directly disrupt AD by interfering with the decomposition of organic matter. They hinder the microbial processes and enzymes responsible for decomposing waste, thus reducing the efficiency of digestion. In contrast, indirect inhibitors such as volatile fatty acids (VFAs), ammonium ions (NH_4_^+^), hydrogen gas (H_2_), and sulfides do not interfere directly but affect AD indirectly. High concentrations of these substances create unfavorable conditions for microorganisms, reducing their activity and the overall efficiency of the process [191]. 

Ammoniacal nitrogen is a byproduct of the biodegradation of protein-rich substrates and exists in two forms: ammonium ion (NH_4_^+^) and free ammonia- FAN (FAN, NH_3_). Although both forms, collectively referred to as Total Ammonia Nitrogen (TAN), can contribute to the inhibition of microbial processes [231], free ammonia (FAN) emerges as a particularly potent inhibitor in AD when its concentration surpasses specific thresholds. Several investigations focusing on the impact of biochar on AD and NH_3_ inhibition suggest that biochar can successfully alleviate NH_3_ inhibition, leading to a shorter lag phase and increased CH_4_ production compared to control tests. According to Su et al. [185], the addition of biochar can mitigate inhibition in food waste AD [232] particularly when the TAN is around 1500 mg/L. Mumme et al. [233] found that biochar derived from wheat husks and paper sludge could mitigate moderate NH_3_ inhibition (2.1 g TAN/kg). Lü et al. [228] reported that biochar can even sustain AD under conditions of high TAN stress of up to 7 g-N/L. While these results suggest that biochar has a positive impact on the inhibition by ammonia, there is no clear consensus on the proposed mitigation pathways, including:

- Cation exchange capacity [190,219],
- Chemical and/or physical sorption, surface functional groups [182,190,219],
- Promotion of direct interspecies electron transfer (DIET) [229,234],
- Immobilization of microorganisms [185,228].

As a result, depending on the characteristics of both biochar and substrate, as well as the AD system parameters, the biochar could well add value to NH_3_ alleviation via direct (CEC, sorption, surface functionality) and/or indirect (DIET and microbial immobilization) factors. Focusing on direct mechanisms, detailed comprehension of the interactions between FAN/TAN and biochar is crucial to identifying its physicochemical features capable of maximizing NH_3_ elimination.

The processes of NH_3_ sorption from wastewater and digestates on biochar have been investigated in the literature [155,228,233,235,236]. High NH_4_^+^ sorption capability, up to 100 mg NH_4_-N/g biochar, has been measured. Physisorption could be facilitated by a high SSA and a developed pore structure, as reported by Yin et al. [224]. However, some investigations have suggested that porosity and SSA may not be the most prominent drivers for NH_4_^+^ sorption [155,235]. For instance, ion exchange involving acidic functional groups on the biochar surface [224,237,238] and CEC [237] may play a significant role in boosting the ammonium sorption capacity of biochar. In particular, Zhang et al. [239] found that biochar developed from the corn-cob pyrolysis at 400 °C exhibited superior NH_4_^+^ sorption performance compared to that developed at 600 °C due to the availability of more acidic functionalities. Therefore, identifying the optimum pyrolysis temperature and other monitoring settings is essential to enhance the biochar adsorptive performance.

The aromatic structure of biochars enables interaction of the sorption mechanism, facilitated by the presence of -OH and -COOH moieties [240]. Tan et al. [241] emphasized the importance of functional groups over the pore size in biochar sorption. However, it has been reported that biochar sorption of VFAs and acidity attenuation enhanced CH_4_ production [242,243]. A direct correlation was identified between SSA and NH^+^_4_-N sorption in biochar [215]. However, Xu et al. [244] demonstrated that 1 g of biochar sorbed 25 mg of NH^+^_4_ and 50 mg of VFAs in AD of pig carcasses.

### 4.5. Effect of Biochar on Biogas Upgrading

Raw biogas produced from AD is typically composed of 50 to 70% *v*/*v* CH_4_ and 30 to 50% *v*/*v* of CO_2_, along with minor constituents such as water vapor, H_2_S, NH_3_, O_2,_ and N_2_ [245]. Upgrading and purifying biogas is essential to meet engine and pipeline standards, although this involves energy and economic costs that can reach up to 55% of the overall CH_4_ production cost [182,246]. So far, conventional technologies involve water treatment, cryogenic separation, physisorption/chemisorption, and membrane separation [247,248]. Recently, biochar has been investigated for its potential as a sorbent for CO_2_ and H_2_S in both in-situ and ex-situ applications.

Given the acidic nature of CO_2_, an increase in basicity on the surface of carbon-based materials promotes CO_2_ uptake due to acid-base reactions [249,250]. As a result, biochar basicity plays a crucial role in CO_2_ uptake by adjusting its interactions with acidic compounds. Chemical alterations can enhance alkalinity, thereby improving CO_2_ uptake and selectivity by creating basic sites [249,251]. Similarly, the sorption of H_2_S from biogas is attributed to the biochar alkalinity, with the more basic biochar demonstrating superior sorption capabilities for this acidic gas [252].

Several investigations [182,188,190,219] have examined the feasibility of in-situ biogas upgrading by adding biochar into the reactor to increase the methane concentration up to gas pipeline standards. In particular, Shen et al. [219] investigated the feasibility of capturing CO_2_ with corn stover biochar during thermophilic AD of waste-activated sludge. Compared to the control, the biochar increased CH_4_ content to 88.5–96.7%, with a CO_2_ reduction of 54.9–86.3%, and maintained an H_2_S concentration of less than 5 ppb, using a concentration of biochar of 1.82–3.64 g/g TS sludge, respectively. The authors suggested that the high porosity of biochar, the substantial SSA, and the abundance of basic and hydrophobic sites might all assist with boosting CO_2_ elimination. In a study by Shen et al. [182], two different woody biochars were introduced into the AD process of waste-activated sludge under both mesophilic and thermophilic conditions. Notably, the addition of biochars led to a significant increase in the average CH_4_ content in the biogas (92.3% under mesophilic and 79.0% under thermophilic conditions), compared to the control (66.2% for mesophilic and 32.4% for thermophilic conditions). This increase in CH4 content led to a corresponding reduction in CO_2_ emissions. The authors attributed these improvements to the specific qualities of biochars, including their high surface area and porosity, chemical stability, and alkaline nature. The study conducted by [188] investigated the overall effect of particle size and dosage of biochar derived from walnut shells on the AD of food waste under mesophilic and thermophilic conditions [188]. Compared to the control test, the authors reported a higher CO_2_ elimination of 61.0% for the biochar with the smaller particle sizes compared to the coarse ones (51.0%); this improvement was attributed to the high SSA and ash percentage. Nevertheless, other studies [182,219] reported a decrease in methane production with higher biochar dosages, suggesting a potential inhibition by elevated cations released from the biochar. In a two-stage digester, Shen et al. [190] investigated the impact of two biochars derived from pine wood and maize stover in the AD of waste-activated sludge. The authors reported higher average CH_4_ contents (81.0–88.6% for maize stover biochar and 72.1–76.6% pine wood biochar), compared to the control (approximately 70.0%). They attributed this enhancement to the discharge of base cations by biochars, aiding in the chemical sorption of CO_2_ and promoting its adsorption through bicarbonate/carbonate salt formation. In addition to CO_2_ adsorption on biochar, the predominant CH_4_ production relies on enhanced syntrophic synergy between organic acid-oxidizing bacteria and CO_2_-reducing methanogens [183,253]. This reinforces the critical importance of effective interspecies electron transfers. Further research to explore the promising results of biochar in-situ upgrading for biogas would be highly advantageous.

Recent noticeable trends involve the utilization of biochar and other carbon adsorbents for capturing CO_2_ from diverse exhaust gas streams [254,255,256]. Numerous studies [240,249,257,258,259,260,261,262,263] have extensively investigated the CO_2_ and H_2_S sorption capabilities of various biochars for ex-situ biogas upgrading and purification. The adsorption capacities for both CO_2_ and H_2_S vary widely, with values ranging from 0.4 to 2.3 mmol/g and 0.2 to 19.1 mmol/g, respectively. Most research on CO_2_ retention focuses on the properties of various biochars, eventually activated, rather than on the composition of the biogas itself. Sethupathi et al. [262] evaluated the uptake of CH_4_, CO_2,_ and H_2_S in a synthetic biogas stream using four biochars derived from perilla leaf, soybean stover, Korean oak, and Japanese oak in fixed bed continuous adsorbers. They found that the biochars could only adsorb CO_2_ and H_2_S, with H_2_S and CO_2_ exhibiting an adsorption ability of 0.208 mmol/g and 0.126 mmol/g, respectively. Creamer et al. [263] investigated the ability of biochar derived from bagasse to adsorb CO_2_. Biochar produced at 600 °C with a high SSA (388 m^2^/g) adsorbed CO_2_ at an amount of about 73.55 mg/g, while biochar produced at 300 °C with a lower SSA (5.2 m^2^/g) had significantly lower CO_2_ adsorption capacity (19.82 mg/g). This highlights the critical role of SSA in capturing and sequestering CO_2_. According to Creamer and Gao [264], physical sorption is the major pathway for CO_2_ capture by biochar. The authors noticed the significance of a greater SSA [265], and a suitable pore size (0.5–0.8 nm) [266], along with Van der Waals and electrostatic interactions. Nevertheless, the chemical characteristics of biochar can also affect CO_2_ uptake due to the availability of basic functional groups on its surface or metals of a basic nature, hydrophobicity, and non-polarity [256]. In a relevant study, Xu et al. [267] observed that the CO_2_ uptake by three biochars during a batch experiment was influenced by the presence of Ca, Fe, K, and Mg, in addition to physical sorption. They further noted that activating the biochar and applying surface treatments could enhance the SSA for physisorption and increase the number of surface functionalities and metal oxides for chemisorption, resulting in impressive CO_2_ sorption capabilities ranging from 5.0 to 7.4 mmol/g [268]. In contrast, Sahota et al. [252] reported significant achievement in removing H_2_S from biogas using biochar derived from leaf waste, achieving an 84.2% elimination percentage. Moreover, another study [240] achieved an impressive 98% H_2_S elimination, equivalent to 8.02 mmol/g, using a biochar. These findings suggest that the carboxyl and hydroxide radical groups in the biochar were responsible for the uptake of H_2_S. In addition, in a specific case, biochar derived from AD digestate demonstrated its effectiveness in adsorbing H_2_S from synthetic biogas [257]. This high efficiency was attributed to the presence of ash, porosity, and aromatics in the biochar. Unlike CO_2_ adsorption, which primarily involves physical mechanisms, the adsorption of H_2_S onto the biochar surface was found to involve various chemical pathways [269].

In addition, CO_2_, H_2_S, and NH_3_, siloxanes present another significant biogas pollutant. These organometallic compounds, featuring “Si-O-Si” bonds, are present in substrates such as municipal sewage sludge or organic municipal solid waste, because of the use of silicon-containing chemicals [270]. Owing to their low water solubility, siloxanes remain bound to sludge flocs and organic matter, and larger siloxane molecules break down into shorter or volatile compounds that are eventually released into biogas [271]. Volatile CH_3_-siloxanes are the most prevalent siloxanes in AD biogas, and their chemical and thermal stability poses a challenge for degradation [272]. To facilitate broader applications, it is imperative that biogas be free of siloxanes. Consequently, adsorption techniques are highly recommended for siloxane removal due to their cost-effectiveness and ease of implementation [270,272].

Activated carbon is the predominant adsorbent used for eliminating siloxanes in biogas purification processes [271,273]. However, biochar has emerged as a potential alternative due to its unique properties. A biochar derived from wood waste has shown its capacity to adsorb octamethylcyclotetrasiloxane from 3.5 to 4.4 mg/g. To enhance the sorption capability of biochar, researchers have proposed activation through chemical or physical methods, as the pore volume and specific surface area (SSA) of biochar play a significant role in the uptake of octamethylcyclotetrasiloxane [274]. In a study focused on removing hexamethyldisiloxane from biogas, Meng et al. [137] investigated the use of coconut shell-based biochar. The untreated biochar exhibited an uptake of 223.3 mg/g, while the modified biochar with Fe_3_O_4_ showed an augmented uptake value of 356.4 mg/g. The enhanced sorption of Fe_3_O_4_^−^-modified biochars were strongly associated with their developed porosity and SSA achieved through the modification process. Moreover, it was observed that the modified biochars demonstrated recyclability without a significant decline in their sorption efficiency [137]. This finding indicates that biochar is a promising sorbent for siloxane elimination in biogas treatment. Considering the presence of diverse siloxane compounds, the development of customized biochars holds the possibility of further enhancing their sorption capabilities.

Papurello et al. [136] proposed the implementation of a biochar-based biogas cleaning process at a pilot AD plant to remove impurities from the biogas intended for solid oxide fuel cell (SOFC) utilization. Their study demonstrated the successful sorption of sulfur compounds, including H_2_S (1.05 mg/g), C_4_H_10_S (1.05 mg/g), and hexamethylcyclotrisiloxane (1.28 mg/g), onto biochar. Additionally, the biochar effectively adsorbed carbonyl compounds such as 2-butanone, terpenes like p-cymene and limonene, as well as aromatic compounds like toluene [136]. These promising findings, showcasing the efficacy of biochar in a practical AD plant for CH_4_ purification, emphasize the need for further research on potential applications of biochar.

## 5. Economic and Environmental Analysis of Biochar Utilization in AD

In recent years, the international biochar sales have increased significantly. Predictions indicate that by 2021 and 2026, biochar sales are expected to reach US$ 1.85 billion and US$ 3.99 billion, respectively [275]. With the increasing demand for renewable energy, the worldwide biochar market’s income is projected to exceed US$ 6.3 billion by 2031 [276]. Until now, scaling up AD combined with the pyrolysis process poses challenges in terms of techno-economic viability. While numerous studies have demonstrated the feasibility of incorporating biochar in AD and CH_4_ purification, and life cycle assessments (LCAs) have supported the use of these additives, there is a notable absence of techno-economic assessments (TEAs) for AD in combination with thermochemical processes for digestate treatment and valorization. It is imperative to conduct TEAs to confirm the system’s viability. Additionally, addressing the cost implications of drying the highly humid digestate, a necessary step to prepare it for biochar production via pyrolysis is crucial. These economic considerations will provide a comprehensive understanding of the overall feasibility and sustainability of this integrated approach.

**Figure 5:**
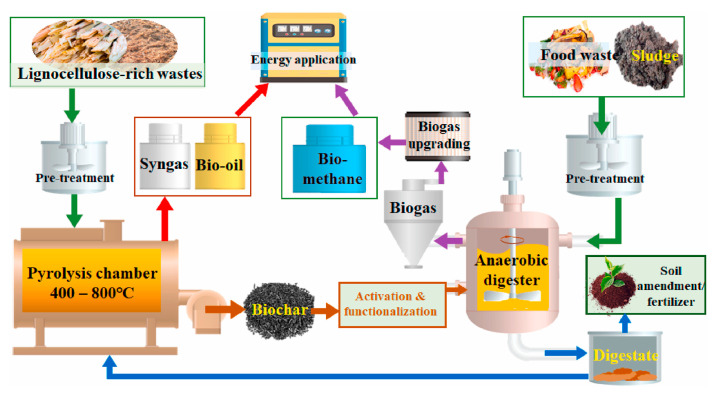
Combined production of biochar and renewable energy from waste and via anaerobic digestion and pyrolysis. (Reprinted from [18], with permission from Elsevier).

The Life Cycle Assessment (LCA), as defined by the International Organization for Standardization (ISO), is a recognized approach involving the collection and analysis of data on the inputs, outputs, and potential environmental impacts of a product system over the course of its entire life cycle. The environmental viability of this approach can be evaluated by combining AD with a thermochemical method for producing biochar from digestate. The integration of AD and pyrolysis, as shown by LCA studies, provides a practical solution for enhancing energy and nutrient recovery through CH_4_ production (Figure 5). Additionally, the outcomes of LCA are significantly influenced by various scenarios for biogas utilization, such as transportation fuel, energy, and power production, and residential cooking [277], highlighting the importance of evaluating the entire integration process of AD and pyrolysis rather than focusing on individual processes. Wang et al. [278] conducted an LCA to assess the environmental impact of municipal solid waste (MSW) recovery through different routes, including AD, pyrolysis, and pyrolysis-AD. The study evaluated various environmental indicators such as acidification, eutrophication, ecotoxicity, climate warming, ozone layer depletion, and respiratory impact. The results showed that the AD-pyrolysis route had the lowest overall environmental footprint among the assessed routes. This was attributed to the higher energy generation and lower emissions from solid digestate in the AD-pyrolysis process [278]. Combining continuous co-digestion (chicken manure and maize stover) AD with pyrolysis has been shown to improve energy efficiency. A study demonstrated that the combination of AD and digestate pyrolysis resulted in an increase in energy return from 48% to 85% [279], indicating that the integration of these processes enhances the overall energy recovery and utilization of the co-digestion system.

According to the TEA conducted by Lin et al. [280], for a thermophilic solid-state AD plant with a capacity of 20,000 metric tons per year, the projected total funding required was $256 per metric ton, while the total annual income was estimated to be approximately $50 per metric ton of capacity. The TEA considered the utilization of biogas for combined heat and power generation. The study found that the majority of the net external energy input was consumed during the process of drying the digestate, which accounted for 63% of the total energy usage. However, the purchase of the digestate revealed that the drying process was essential for the viability of the overall process [280]. The authors emphasized that it is crucial to assess various methods of valorization to increase the selling price of the digestate, [280]. The TEA provides important insights into the economic aspects of the thermophilic solid-state AD process and highlights the need to evaluate different approaches for enhancing the value of the digestate. The economic viability of AD has been established through sensitivity analysis, with the sale price of digestate identified as a crucial factor [280,281,282]. Thermochemical processes can represent crucial technologies by exploring new possibilities for digestate management. One such possibility is the conversion of digestate into biochar, offering various potential uses. Using biochar as a soil fertilizer eliminates potential disease problems and increases the profitability of the process. A TEA by Haeldermans et al. [283] for large-scale production of biochar through conventional pyrolysis from various wastes (3 tons/h) indicates the viability of the plant. The minimum sales cost of biochar ranges from 436 to 863 euros per ton. In the case of biochar produced from orchard biomass using mobile pyrolysis technology, the estimated cost ranges from 449 to 1847 USD per ton, with a 90% probability of 571 to 1455 USD per ton [284]. In the fast pyrolysis of date palms, biochar accounts for 80% of revenues from the sale of pyrolysis products (char, oil, and gas). Considering a biochar price of USD 1200 per ton, biochar accounts for almost 40% of the total energy production. The economic evaluation of fast pyrolysis is particularly sensitive to the trade prices of the products considered [285].

In the specific context of biochar production, the main factors that impact pyrolysis yield, bio-oil molecular weight, and biochar production, include ash and lignin concentrations, as well as the O/C ratio [286]. Li et al. [287], using a regression model on 346 lignocellulosic feedstocks, found that higher ash concentration increases biochar output by 12.5% to 15.5%, simultaneously reducing bio-oil production and subsequent revenues from bio-oil commerce, making pyrolysis economically advantageous. Lower ash content and higher O/C ratios of raw materials lead to higher biofuel yields, enhancing economic performance. In the context of the pyrolysis process for the biochar production from lignocellulosic raw materials, the initial investment is distributed as follows: 43% for pre-treatment and pyrolysis, 35% for H_2_ generation, and 22% for cooling and fractionation processes [287]. Due to higher disposal and pre-treatment costs for straw biomass, accounting for 32% and 34% of the total operational expenses, respectively [288], the average operating cost ranges from 0.68 Euro/L for woody biomass to 0.86 Euro/L for straw biomass. Economic analyses from two studies [289,290] suggest that a biochar market price of 470 Euro/t is necessary to cover the investment and operating expenses associated with biochar production. Expanding the viability and applicability of AD to unconventional substrates incurs additional expenses, which may be offset by the potential increase in electric energy provided by higher CH_4_ output. Inorganic and biological supplements, such as iron, micronutrients, and ash, are commonly used to enhance CH_4_ production by reducing inhibition and facilitating organic material solubilization. However, the use of supplements in AD incurs costs of 3.60–4.10 Euro/L enzyme and 13–16 Euros/L nutrients [134,291]. The manufacturing costs of biochar can be as low as 0.2 to 0.5 USD/kg, depending on the raw material and pyrolysis method. This makes biochar less expensive than granular activated carbon, which can range from 0.6 to 20 USD/kg. AD of waste biomass and pyrolysis of digestate has the potential to yield higher net electricity output [292] compared to AD alone, resulting in economic and environmental benefits [293]. However, there is still some uncertainty regarding the balance between the entry costs of biochar incorporation and the energy output efficiency of AD. Zhang et al. [294] explored the addition of woody biochar to enhance thermophilic anaerobic digestion of food waste and concluded that biochar addition has the potential to be cost-effective.

When evaluating the viability of exploiting digestate for biochar production, it is essential to conduct a comprehensive TEA. This analysis has to include not only the drying cost of digestate, but also all expenses associated with AD, pyrolysis, and biochar utilization. It serves as a key factor in determining the economic viability of the integrated approach and establishes the conditions under which it can be financially viable.

In this context, reducing the water content of digestate is crucial for its conversion into biochar by pyrolysis. Pyrolysis, characterized by thermal decomposition, relies on high temperatures, and excess moisture can considerably hamper its efficiency. Therefore, it is imperative to consider at costs associated with the drying phase, which is essential for biochar production from digestate. Drying digestate requires energy, whether by conventional methods such as air drying or by advanced alternatives such as solar or thermal drying. The choice of energy source and the efficiency of the drying process directly impact the overall cost. This issue becomes particularly relevant when processing large quantities of digestate, as prolonged drying times progressively extend the production schedule and associated costs. In addition, establishing the essential drying infrastructure, including drying beds, drying ovens, or solar drying systems, involves both initial investment and ongoing maintenance costs. Environmental considerations are closely linked to the choice of energy source for drying. For instance, the use of fossil fuels in the drying process would amplify the carbon footprint of the entire operation.

In summary, the drying cost plays a key role in evaluating the economic feasibility of the entire AD biochar production system. If drying costs become excessive, they risk overcoming the benefits of biochar production from digestate. Consequently, exploring energy-efficient drying methods and exploiting renewable energy sources emerge as strategies to reduce costs and decrease the environmental impact of the drying process, thus improving the entire sustainability of the system.

## 6. Future Prospects and Emerging Challenges

There is an increasing interest in exploring the combined utilization of biochar and AD to address the specific issues related to the AD system. The future prospects of using biochar in AD are promising, showing the potential to significantly improve CH_4_ production rates and process stability. As more research is conducted, it is anticipated that optimized biochar types and their optimal concentrations in AD systems should be determined, leading to increased efficiency and effectiveness in CH_4_ production. Additionally, the combination of biochar and AD offers a valuable solution for sustainable waste management, reducing the environmental impact of various organic waste, such as agricultural residues or municipal solid waste. Incorporating biochar-enhanced AD into renewable energy systems could provide a consistent stream of clean energy while decreasing reliance on fossil fuels. Furthermore, by capturing and storing carbon through biochar production and increasing sustainable energy production in the form of biogas, this approach can contribute to reducing global climate change.

However, the development and deployment of biochar-enhanced AD face new barriers. Scalability is a major challenge, as it is crucial to create cost-effective technologies for manufacturing biochar from digestate and incorporating it into an AD system that can be adapted to varied scales, from small farms to huge industrial units. As technology progresses, academics and practitioners must focus on developing systems that are simple to deploy in a variety of scenarios.

Through this review, it has been demonstrated that biochar plays an essential role in enhancing electron transfer mechanisms in AD processes. Its redox properties, influenced by surface functional groups, metals, and metal oxides, have a significant impact on its extracellular electron conductivity (EEC), facilitating electron transfer., Although the EC of biochar is lower than that of other conductive materials, it effectively promotes DIET, improving the efficiency of methane production and the dynamics of the microbial community. In addition, its buffering capacity stabilizes pH levels and prevents the accumulation of volatile fatty acids, further enhancing AD performance. In addition to its EC and redox properties, high SSA and porosity of biochar contribute to its positive impact on methanogenesis. The role of biochar in attenuating ammonia inhibition and enhancing biogas generation, as well as the uptake of NH_3_, H_2_S, and CO_2_ from biogas, should also be investigated.

In the future, research should focus on several promising directions:

- Tailoring redox properties and EC of biochar: Developing innovative methods to tailor the redox properties and EC of biochar, potentially through the introduction of specific functional groups or improved activation processes, could further enhance its performance in AD systems.
- Microbial interactions and syntrophic partnerships: Investigating how biochar interacts with various microbial communities in AD reactors and its effects on specific syntrophic partnerships is crucial and could lead to a better understanding of the role of biochar in the evolution of microbial dynamics.
- Feedstock influence on biochar properties: Exploring and understanding how different feedstock sources for biochar production influence its properties and performance in AD could lead to feedstock-specific recommendations for optimizing AD processes.
- Understand the various functions of biochar to counter NH_3_ inhibition, optimize its properties for efficient NH_3_ removal by studying interactions with FAN/TAN, and examine the impact of its pore size and SSA on NH_3_ sorption during anaerobic digestion.
- Effectiveness of biochar in removing other contaminants from biogas: Investigating the ability of biochar to remove CO_2_ and other impurities is crucial to assess its overall potential for biogas cleanup and upgrading.
- Competitive sorption: Examining how CO_2_, H_2_S, and NH_3_ compete with each other for adsorbing on the sites of adsorption on biochar surfaces could help determine the efficiency of the biochar in simultaneously eliminating these contaminants. This research can identify potential modifications for enhanced selectivity and capability.
- Water vapor impact: Investigations on how the presence of water vapor in biogas impacts the sorption of CO_2_, H_2_S, and NH_3_ on biochar is essential.
- CH_4_ loss: Future research should focus on understanding how methane fixes onto biochar and investigating methods to prevent or minimize this process. This will enable a greater quantity of CH_4_ to be maintained in purified biogas.

Another challenge is the standardization of this integrated process. To ensure the safety, effectiveness, and optimization of biochar-enhanced AD, it is essential to establish standardized guidelines for the production and application of biochar. This would facilitate comparability across studies, enabling researchers to build on each other’s work and continuously improve the technology.

Public awareness and acceptance of biochar-enhanced AD are also vital for widespread adoption. Efforts aimed at communicating the benefits and potential risks of this technology to stakeholders and the general public will be crucial in overcoming existing skepticism and promoting support for the use of this technology.

Developing appropriate regulatory frameworks and policies to support the use of biochar in AD is another essential aspect. Regulatory measures will be needed to ensure environmentally responsible practices and foster the technology’s growth across various sectors.

Lastly, understanding the long-term impacts of using biochar in AD is critical. Comprehensive studies investigating the effects on soil health, crop productivity, and environmental sustainability will be necessary to gain a holistic understanding of the technology’s potential benefits and drawbacks. Ultimately, this will inform best practices and guidelines for its use.

## 7. Conclusions

The AD process stands out as a straightforward technology for treating organic waste, offering significant environmental, energy, and economic potential. Various pathways facilitated by different types of microorganisms enable the conversion of organic waste into biogas. Key operational parameters such as temperature, moisture content, pH, organic loading rate, and C ratio influence process phases, affecting overall efficiency.

A substantial amount of digestate is generated annually from AD, necessitating careful management for agronomic purposes. Factors governing digestate storage duration include local regulations, weather conditions, soil type, crop cycles, and operational procedures. Improper handling can lead to methane and ammonia emissions, impacting fertilizer quality, greenhouse gas emissions, public perception, and economic viability.

Considering ecological impacts, organic content quality, disposal costs, and nutrient availability, land application alone is insufficient. Therefore, exploring alternative valorization methods for digestate, particularly through pyrolysis for biochar production, is essential. Pyrolysis technology optimizes AD by converting digestate into biochar and enhancing overall sustainability through additional oil and syngas production and higher carbon stability. While conventional biochar has been extensively researched for soil improvement and contaminant remediation, studies on digestate-derived biochar remain limited but promising. Biochar derived from digestate holds potential as a soil conditioner, contributing to carbon sequestration and environmental benefits such as contaminant remediation. It improves microbial growth, system buffering capacity, and Direct Interspecies Electron Transfer (DIET), leading to increased methane production.

Future research should focus on fully characterizing digestate-derived biochar and its application in AD systems. Understanding how biochar characteristics impact AD efficiency, including surface area, pore structure, functional groups, elemental composition, and ash content, is crucial. Mechanisms such as ammonium sorption and microbial pathways need further exploration. Additionally, biochar shows promise in upgrading biogas by adsorbing impurities like CO_2_, H_2_S, NH_3_, and siloxanes. Integrating digestion with pyrolysis presents an effective solution to challenges associated with digestate management.

In conclusion, utilizing digestate-derived biochar in AD systems or as a biogas purification material can advance circular-economy-driven processes. Further research is necessary to assess the technical, financial, and environmental efficiencies of alternative process designs integrating digestion with pyrolysis.

## Figures and Tables

**Table 1:** Examples of the dominant microbial functional groups involved in the anaerobic digestion process [23].

Microbial Groups	Substrates	Products	Examples
Hydrolytic/fermentative bacteria	Cellulose, hemicelluloses	Cellobiose, hexoses, pentoses, acetic acid, ethanol, CO_2_	*Phylum Firmicutes and Bacteroidetes.Genus Acetivibrio, Bacteroides, Clostridium, Ruminococcus, Thermotoga*
	Protein	Peptides, amino acids	*Phylum Proteobacteria*
Syntrophs	Propionate, butyrate,	Acetate, H_2_, CO_2_	*Syntrophomonas, Smithella, Synergistes*
Syntrophic acetate-oxidizing bacteria	Acetate	H_2_, CO_2_	*Clostridium ultunense, Thermotoga lettingae, Thermacetogenium phaeum*
Homoacetogens	H_2_, CO_2_	Acetate	*Acetobacterium*
Acetotrophic methanogens	Acetate	CH_4_, CO_2_	*Methanosaeta, Methanosarcina*
Hydrogenotrophic methanogens	H_2_, CO_2_	CH_4_	*Methanosarcina, Methanobacteriales, Methanospirillum, Methanoculleus*

**Table 2:** Benefits and drawbacks of mesophilic and thermophilic bacteria [27,60].

	Mesophilic	Thermophilic
Benefits	Works with tough bacteria that can tolerate more environmental changes. The system is more reliable and straightforward to maintain. Bacteria [61]: *phyla Firmicutes, Proteobacteria, Bacteriodetes and Chloroflexi.* Lower energy costs.	The needed retention time is decreased as the temperature rises. Bacteria [61]: *Firmicutes, Proteobacteria, Chloroflexi and Actinobacteria.* Increased pathogen elimination.
Drawbacks	Increased retention time. Decreased biogas production.	Because the microbial population is less diversified, the process is less steady; the system is more difficult to maintain. Extra energy input is required for heating.

**Table 3:** The outcomes of various studies on co-digestion of OMSW and other types of waste.

AD	Substrates	Dry Weight Ratio	C/N Ratio	CH_4_ Yield (LCH_4_/gVS)	Refs.
Thermophilic batch	OMSW:Sludge	2:1	14.19	0.14	[88]
Two-stage: thermophilic-mesophilic	OMSW:Sludge	2:1	14.19	0.18	
Single-stage: mesophilic	Food waste:Sludge	1:9	5.97	0.18	[89]
		3:7	6.99	0.21	
		1:1	8.9	0.32	
		7:3	11	0.33	
		9:1	14.7	0.34	
Mesophilic batch	Food waste:Cattle manure	2:1	15.8	0.38	[90]
Two-phase	OMSW:Cow manure	10:1	20	0.10	[91]
Mesophilic batch	OMSW:Sludge	1:34	17.68	0.15	[92]
		1:19	20.55	0.20	
Singles stage: mesophilic	Food waste:Sludge	1:2.4	7.1	0.30	[93]
		1:0.9	10.2	0.35	
		1:0.4	11.4	0.40	
Mesophilic	OMSW		14.1	0.38	[94]
	OMSW:Vegetable oil	5:1		0.69	
	OMSW:Animal fat	5:1		0.50	
	OMSW:Cellulose	5:1		0.25	
	OMSW:Protein	5:1		0.28	

## Data Availability

Not applicable.

## Supplementary Materials

The following supporting information can be downloaded at https://www.mdpi.com/article/10.3390/ma17143527/s1, Table S1: Characteristics of digestate obtained from various sources of raw material [121,124,152,153,156,162,163,164,165,166,168,169,170,174,175,293,295,296,297,298,299,300,301,302,303,304,305,306,307,308,309,310,311,312]. Table S2: Biochar in AD: Source, conditions, and results effects [98,132,182,190,195,196,201,219,226,228,243,313,314,315].

## Abbreviations

AD	anaerobic digestion	IET	interspecies electron transfer
BOD	biological oxygen demand	LCFA	Long-chain fatty acids
BC	biochar	LCA	life cycle assessment
BMP	biochemical methane potential	MSW	municipal solid waste
COD	chemical oxygen demand	MR	microwave irradiation
CEC	cation exchange capacity	OMSW	organic municipal solid waste
DIET	direct interspecies electron transfer	ORP	oxidation-reduction potential
DOC	dissolved organic carbon	OLR	organic loading rate
DM	dairy manure	SSA	porosity, specific surface area
EC	electrical conductivity	TEA	techno-economic assessment
EAC	electron-accepting capacity	TS	total solids
EDC	electron-donating capacity	TAN	total ammonia nitrogen
FAN	free ammonia nitrogen	VFA	volatile fatty acids
HRT	hydraulic retention time	VS	volatile solids
HC	hydrochar	WAS	waste activated sludge
