# Supplementary material for: Integration of Digestate-Derived Biochar into the Anaerobic Digestion Process through Circular Economic and Environmental Approaches—A Review
Source: Materials (Basel). 2024 Jul 16;17(14):3527. doi: 10.3390/ma17143527 (PMC11278828; doi:10.3390/ma17143527)
Supplement: Supplementary file 1 [file materials-17-03527-s001.zip › materials-3086484-supplementary.pdf]

Supplementary Information (SI)

Integration of Digestate-Derived Biochar into the Anaerobic Digestion Process through Circular Economic and Environ-mental Approaches—A Review

Mohamed Zbair[1,2], Lionel Limousy[1,2,*], Méghane Drané[1,2], Charlotte Richard[3], Marine Juge[3], Quentin Aemig[3], Eric Trably[4], Renaud Escudie[4], Christine Peyrelasse[5], Simona Bennici[1,2]

1 Institut de Science des Matériaux de Mulhouse (IS2M), Université de Haute-Alsace, CNRS, IS2M UMR 7361, F-68100 Mulhouse, France ;

2 Université de Strasbourg, France

3 ENGIE, Lab CRIGEN, 4 Rue Joséphine Baker, 93240 STAINS, France

4 INRAE, Univ. Montpellier, LBE, 102 Av. des Etangs, F-11100, Narbonne, France

5 APESA, Pôle Valorisation, 3 Chemin de Sers, 64121, Montardon, France

*Corresponding author: lionel.limousy@uha.fr; +33 (0)3 89 60 87 05

Table S1. Characteristics of digestate obtained from various sources of raw material.

AD feedstocks	AD process	Composition (dry basis) %												Ref.
		Moisture	C	N	P	S	Na	Mg	K	Ca	Fe	Si	Al	
Corn silage, cow manure	Mesophilic	-	52.05	2.33	2.26	0.65	0.58	1.27	1.32	4.1	1.27	-	0.19	[1]
Cow manure, maize silage, grass silage, whole plant silage, cereals	Thermophilic	-	40.3	2.1	0.94	0.3	0.08	0.74	3.67	2.53	0.23	-	0.25	[2,3]
Food waste	Mesophilic	90.2	42.1	5.81	1.97	-	-	0.27	0.62	3.17	0.93	-	-	[4]
Kitchen waste, garden waste	Thermophilic	-	34.3	1.9	0.39	0.2	0.66	0.54	1.8	4.29	0.66	-	0.92	[2,3]
Maize silage, liquid cattle manure,	Mesophilic	93.5	50.5	3.6	1.35	0.5	-	0.94	5.23	2.01	0.3	6.71	0.52	[5]

grass silage														
Activated sludge	-	-	-	-	-	-	-	-	-	-	-	-	-	[6]
Agricultural feedstock	-	59.7	49.24	3.74	-	0.29	-	-	-	-	-	-	-	[7,8]
Agricultural feedstock	-	91.34	44.63	-	-	-	-	-	-	-	-	-	-	[9]
Agro-industrial residues, herbaceous biomass	Mesophilic	-	38.5	1.21	1.82	0.29	0.13	0.85	2.83	3.92	0.19	-	-	[10]
Animal slurry industrial organic waste	-	-	36.63	1.72	-	-	-	-	-	-	-	-	-	[11]
Animal sewage, cow manure, maize triticale silages, cereal bran	Mesophilic	-	43	1.3	-	0.14	-	-	-	-	-	-	-	[12]
Cattail	Thermophilic	-	62.71–63.53	-	0.17	-	-	-	-	0.27	-	-	-	[13–15]
Cattle manure, maize silage	-	-	44	1.7	4.5	0.4	-	-	0.88	-	-	-	-	[16]
Corn silage, manure, vegetable waste	Thermophilic	-	39.8	1.23	0.79	0.29	0.08	0.47	-	2	0.09	-	-	[17]
Corn stalk	Mesophilic	-	38.01	2.05	-	0.62	0.38	0.62	1.25	0.97	-	-	-	[18,19]
Corn straw, sludge, cattle manure	-	-	29.64	2.38	-	0.54	-	-	-	-	-	-	-	[20]
Cow manure	-	85–89	39.11	1.94	-	0.68	-	-	-	-	-	-	-	[21]

Cow manure/food waste	-	24.5	37.8	1.09	0.59	-	-	-	0.32	-	0.87	-	-	[22]
Food waste	Mesophilic	-	42.1	5.81	-	0.91	-	-	-	-	-	-	-	[23]
Food waste	-	-	43.5	1.92	-	0.11	-	-	-	-	-	-	-	[24]
Groats, olive oil cake, silage of triticale, chicken manure	Mesophilic	-	42.5	1.4	-	0.14	-	-	-	-	-	-	-	[12]
Herbaceous biomass, agro-industrial residues	Mesophilic	76.2	46.7	1.2	-	0.5	-	-	-	-	-	-	-	[25]
Maize silage	Thermophilic	-	46.6	1.1	-	0.08	-	-	-	-	-	-	-	[26]
Municipal solid waste	-	48.2	16.25	0.74	-	0.52	-	-	-	-	-	-	-	[7,8]
Municipal biowaste	-	-	54.5	7.5	-	-	-	-	-	-	-	-	-	[27]
Pig manure	-	-	34.08	4.26	4.09	1.89	0.23	1.32	0.8	9.93	0.79	1.06	0.29	[28]
Pig manure, cow manure, maize, triticale silages, cereal bran	Mesophilic	-	43	1.3	-	0.14	-	-	-	-	-	-	-	[29]
Pig slurry, olive pomace, maize silage, sorghum silage, onion scraps	Mesophilic	-	42.52	1.79	-	-	-	-	-	-	-	-	-	[30]
Sewage sludge	-	4.66 (Dried)	34.78	5.04	-	1.15	-	-	-	-	-	-	-	[31]
Sewage sludge	-	5.72 (Dried)	38	4.29	-	-	-	-	-	-	-	-	-	[32]
Sewage sludge	Mesophilic	83.5	32.7	5.1	4.58	1	0.16	0.32	0.28	3.02	1.58	1.94	-	[33]
Vegetable, garden,	Thermophilic	-	29.5	2	2.6	0.3	0.5	0.8	0.7	4.3	3	7.1	-	[34]

fruit (VGF)														
Municipal solid waste	Thermophilic	-	24.1	1.5	0.7	0.2	0.9	1.4	1.6	10.4	3.2	10.2	-	

Table S2. Biochar in AD: Source, conditions, results effects.

Biochar source	Pyrolysis Temperature (°C)	AD process conditions	Principal results on biochar effects in AD	Ref.
**Fruit woods**	800 °C	Mesophilic batch wet AD at 35 °C<br><br>Substrate: glucose, yeast extract	• The addition of 0.5–1 mm biochar (10 g/L) to digesters enhanced performance by shortening the lag phase and increasing the maximum methane production rate.<br>• 75 μm biochar further reduced the lag phase by 38.0% and increased methane production by 70.6% at 6 g/L glucose loading.<br>• Biochar had an impact on the production and degradation of VFA.<br>• Fingerprint and sequencing analysis revealed a higher proportion of Archaea in biochar-added conditions.<br>• *Methanosarcina* was predominant in larger 2-5 mm biochar particles.<br>• *Methanosaeta was* enriched in loosely-bound fractions with all-size biochar particles.<br>• Biochar did not significantly adjust system pH or acted solely as a cell growth carrier.<br>• Biochar was found to benefit functional microbes through different mechanisms, including promoting biofilm growth and facilitating direct electron or hydrogen transfer between syntrophs and methanogens.	[35]
**Saw dust**	500 °C	Thermophilic batch wet AD at 55 °C<br><br>Substrate: food waste and waste activated sludge in co-digestion mode	• Co-digestion of food waste (FW) and waste activated sludge (WAS) with biochar enhanced methane production, especially at high organic loading.<br>• Biochar improved buffer capacity, preventing sharp pH decreases due to biochar's alkalis.<br>• At high organic loading, VFAs accumulated rapidly, causing pH imbalance and acidification in control groups. Biochar prevents the pH drop due to its buffering capacity.<br>• Biochar's porous structure allowed for quick microbial attachment and acclimation, reducing lag time and hastening the process.<br>• In the presence of VFA accumulation, control groups showed lower methane production due to inefficient H_2 transfer.<br>• Biochar facilitated efficient electron exchange between bacteria and methanogens, similar to DIET.<br>• Microbial analysis confirmed selective succession of microbes, improving syntrophic metabolism in the presence of biochar.	[36]
**Fruit woods**	800–900 °C	Mesophilic batch wet AD at 35 °C; ISR: 1:6<br><br>Substrate: glucose, yeast extract.	• The addition of biochar resulted in a shortened lag phase during AD and increased methane production rate, even under high ammonium stress.<br>• Small particle size biochar promoted VFAproduction.<br>• Biochar particle size influenced microbial accessibility.<br>• This information suggested a DIET between syntrophs and Methanosaeta .	[37]
**Dairy manure**	350 °C	Batch AD; psychrophilic at 20 °C; mesophilic at 35 °C; thermophilic 55 °C	• The addition of biochar enhanced methane production and shortened the lag phase across all temperatures.<br>• Acetic acid and propionic acid were identified as the dominant VFAs in all experimental sets. The addition of biochar resulted in lower concentrations of total VFAs and propionic acid compared to conditions without biochar.<br>• Biochar facilitated the syntrophic oxidation of propionic acid, which is a limiting step for methanogenesis.	[38]

Biochar source	Pyrolysis Temperature (°C)	AD process conditions	Principal results on biochar effects in AD	Ref.
		Substrate: dairy manure; varied biochar loading	• The alkaline nature of biochar contributed to an increased in pH and total alkalinity, improving reactor stability.	
**Rice straw**	500 °C	Thermophilic operated AD at 55 °C<br><br>Substrate: OFMSW; varied dosage of biochar doped with $FeCl_3$	• Magnetic biochar fabricated with a 3.2 g $FeCl_3$:100 g rice-straw ratio led to an 11.69% increase in methane production compared to the control without biochar. This improvement was attributed to the selective enrichment of microorganisms that participate in AD on the magnetic biochar.<br>• Magnetic biochar fabricated with a 32 g $FeCl_3$:100 g rice-straw ratio resulted in a 38.34% decrease in methane production. This decrease was linked to competition with iron oxide for electrons, negatively affecting the AD process.<br>• High microbial activity and the presence of Fe-reducing bacteria on magnetic biochar indicated potential electron transfer and DIET.<br>• Approximately 25% of the total methanogens were absorbed on magnetic biochar and these could be harvested using a magnet. This presents a potential solution for preventing the loss of methanogens in AD, which is crucial for efficient AD.<br>• The study suggests that magnetic biochar with a specific fabrication ratio, significantly improved methane yield in OFMSW AD by selectively enriching functional bacteria and methanogens..	[39]
**Fruit wood**	800–900	Mesophilic batch wet AD at 35 °C<br><br>Substrate: food waste; ISR: varied; biochar to substrate ratio varied	The addition of biochar resulted in shortened lag phase and an increase in the maximum CH_4 production rate.<br>• Biochar enhanced the degradation rates of dissolved organic matters and VFA.<br>• Biochar immobilized microorganisms and promoted biofilm growth.<br>• Biochar accelerated the development of electrical connections for DIET in co-cultures.	[40]
**Coconut shell**<br><br>**Rise husk**<br><br>**Wood**	450 °C	Mesophilic batch wet AD at 35 °C<br><br>Substrate: citrus peel; ISR: varied; biochar to substrate ratio varied	• The addition of biochar resulted in a shortened lag phase, slight increase in CH_4 production, and an increase in colonies of methanogens.<br>• Biochar improved the efficiency of limonene removal.<br>• Biochar's sorbing properties reduced limonene bioavailability.<br>• Biochar provided a surface for microbial colonization and immobilization.	[41]
**Canola meal**<br><br>**(CBC)**	700-900 °C	Mesophilic batch operated AD at 37 °C<br><br>Substrate: glucose and bio-oil aqueous phase	• ABC-400 and SBC-500 biochars reduced the lag phase and were more effective at promoting methane production compared to GAC.<br>• The EC of the GAC is higher, but the electrical conductivity of the biochars (SBC-500 and ABC-400) remains similar. It is interesting to note that, although GACs have higher EC values, biochars outperform GACs in terms of methane yield. This suggests that factors other than electrical conductivity play an important role in methane production during this process.<br>• Biochars facilitated the reductive transformation of organic contaminants by aiding in electron transfer. This effect was attributed to redox-active structures and phenolic moieties, which act as electron donors.<br>• ABC-400 was particularly effective in biogas production from complex organic waste and showed the best biogas production among the tested biochars.<br>• Concentrations of redox active moieties (RAMs) were higher in intermediate to high-temperature biochars, such as ABC-400. This correlated with increased methane production, suggesting RAMs play a crucial role.	[42]
**Switch grass**<br><br>**(SBC)**	500 °C			
**Ashe juniper**<br><br>**(ABC)**	400-600 °C			
**Pine wood**<br><br>**White oak**	Commercial	Mesophilic and thermophilic operated AD at	• In both mesophilic and thermophilic conditions, P250 and WO250 had similar cumulative biogas production compared to the control.<br>• P500 and WO500 produced less biogas than the control.	[43]

Biochar source	Pyrolysis Temperature (°C)	AD process conditions	Principal results on biochar effects in AD	Ref.
		37 °C and 55 °C respectively<br><br>Substrate: sewage sludge; varied dosage of biochar. P250:inoculum+ substratec+ biochar (2.49 g/g dry matter); P500: inoculum + substrate + biochar (4.97 g/g dry matter); WO250: inoculum + substrate + biochar (2.20 g/g dry matter); WO500:inoculum + substrate + biochar (4.40 g/g dry matter)	• The rate of methane production was generally higher in thermophilic conditions.<br>• Biochar addition improved process stability by increasing alkalinity, although inhibitory effects were observed at high biochar dosage.	
**Pine saw dust**	650 °C	Two phase mesophilic operated AD at 35 °C<br><br>Substrate: food waste	• Biochar significantly increased the maximum production rates of both CH_4 and H_2<br>, and the lag phase was shortened<br>• Biochar enhanced VFA formation in H_2 production phase and VFA degradation in CH_4 production phase.<br>• In hydrogen production phase, biochar likely enhanced biofilm formation, provided temporary nutrients, and buffered pH.<br>• The high surface area (130 m^2/g) of biochar promoted microbial colonization, while volatile matter on biochar surfaces acted as temporary substrates for microbial growth.<br>• Biochar's pH (9.6) buffering capacity maintained stable pH levels, preventing the inhibition of AD by VFA accumulation.<br>• In methane production phase, biochar acted as a microbial carrier, fostering biofilm development and accommodating various microbial populations.<br>• Biochar's porous structure promoted methanogenic biofilm formation, enhancing microbial activity and resulting in faster VFA degradation and methane production.	[44]
**Saw dust**	500 °C	Mesophilic batch operated AD at 35 °C<br><br>Substrate: dewatered activated sludge and food waste; varied biochar particle size and ISR	• Biochar addition reduced the lag phase for methane production by 27.5% to 64.4% compared to the control.<br>• Maximum methane production rate increased by 22.4% to 40.3%.<br>• Optimal results were achieved with a biochar dosage of 15 g/L, even under high organic loads (3 g substrate/g inoculum).<br>• Biochar (pH 9.2) demonstrated a remarkable pH buffering capacity, mitigating the pH decrease caused by VFAs accumulation.<br>• A specific experiment using butyrate as a substrate revealed that under high H_2 partial pressure, syntrophic degradation of butyrate to acetate occurred.<br>• Biochar addition enriched specific microorganisms including *Anaerolineaceae* known for their involvement in syntrophic interactions and *Methanosaeta* associated with DIET.	[45]
**Corn stover**<br><br>**Pine**	Commercial	Two stage semi-continuous thermophilic operated AD at 55 °C<br><br>Substrate: sewage sludge; varied biochar dosage	• Both biochars (corn stover biochar (CSBC) and pine biochar (PBC)) improved AD performance in terms of methane production, process stability, and ammonia-N reduction.<br>• Response Surface Modeling indicated that the percentage of CH_4 was positively associated with Biochar Loading (BCL).<br>• CH_4 production showed a positive correlation with OLR.<br>• Both BCL and OLR were identified as important parameters for optimizing methane production.<br>• Biochar achieves TAN reduction through two primary mechanisms: sorption and precipitation.	[46]

Biochar source	Pyrolysis Temperature (°C)	AD process conditions	Principal results on biochar effects in AD	Ref.
			• Precipitation occurs when magnesium ions (Mg^{2+}) released from biochar ash combine with ammonium (NH_4^+) and phosphate ions (PO_4^{3-}) to form struvite.	
**Vermicompost**<br><br>**(VC)**	500 ℃	Mesophilic batch operated AD at 35 ℃<br><br>Substrate: kitchen waste (KW) and chicken manure (CM)	• The higher buffering capacity of biochar improved CH_4 production.<br>• When VFAs were introduced into biochar, pH decreased more slowly than when they were introduced into VC. This suggests that biochar may be more resistant to pH changes than vermicompost. Biochar contains higher levels of alkali and alkaline-earth metals, such as sodium, potassium, calcium, and magnesium.<br>• The buffering capacity of biochar increased linearly with the proportion of biochar added.<br>• CM digestion did not successfully initiate at a higher organic loading of 50 g TS/kg. However, the addition of 5.0% biochar or VC improved the CM AD performance. .<br>• KW digestion remained unsuccessful, even with VC or biochar increased to 15% and 20%. However, the addition of biochar or VC helped alleviate KW digestion issues (VFAs), with biochar demonstrating better results than VC.<br>• During CM digestion with VC, the average concentration of VFAs was significantly lower with biochar. Additionally, biogas production was delayed by 10 days in CM digestion with VC, along with a rapid decrease in pH. This reflects the critical role of biochar's acid-buffering capacity in improving AD.	[47]
**Corn stover**	600-900 ℃	Thermophilic batch operated AD at 55 ℃<br><br>Substrate: sewage sludge; varied biochar dosage	• Biochar addition led to higher biogas methane concentration and production rates.<br>• The high content of alkali and alkaline earth metals (K, Ca, and Mg) in biochar caused the biochar-amended digesters to have a slightly alkaline pH.<br>• Biochar addition raised the pH in the digesters, promoting hydrolysis and VFA production.<br>• Final pH values remained slightly alkaline (pH > 7.5) after AD, indicating a strong buffering capacity that contributes to process stability.<br>• EC of all digesters increased after AD, particularly in biochar-dosed digesters, likely due to cation release from biochar. This high EC may enhance extracellular electron transfer for methanogenesis.<br>• Biochar addition caused an increase in total organic carbon (TOC) in the sludge, possibly due to the volatile matter in biochar. However, biochar stores this organic carbon in a recalcitrant form, making it resistant to microbial degradation.<br>• The alkaline pH in biochar-amended digesters facilitated the conversion of CO_2 to bicarbonate/carbonate ions. This conversion to bicarbonate/carbonate ions improved buffering capacity, preventing pH drops resulting from VFA accumulation.<br>• Total alkalinity concentrations in biochar-amended digesters were significantly higher than the control. This increase was mainly due to cation release and ammonium formation during AD, maintaining desirable alkalinity levels for the process.<br>• Biochar addition mitigated NH_3 inhibition, enhancing CH_4 production. This is attributed to its large surface area promoting NH_3 adsorption.<br>• Biochar-amended digesters achieved a significant CO_2 removal efficiency ranging from 54.9% to 86.3%.<br>• Biochar-amended digesters had significantly lower H_2S concentration (<5 ppb).	[48]

References

1. Garlapalli, R.K.; Wirth, B.; Reza, M.T. Pyrolysis of hydrochar from digestate: Effect of hydrothermal carbonization and pyrolysis temperatures on pyrochar formation. *Bioresour. Technol.* **2016**, *220*, 168–174, doi:10.1016/j.biortech.2016.08.071.

2. Cao, L.; Wang, J.; Xiang, S.; Huang, Z.; Ruan, R.; Liu, Y. Nutrient removal from digested swine wastewater by combining ammonia stripping with struvite precipitation. *Environ. Sci. Pollut. Res.* **2019**, *26*, 6725–6734, doi:10.1007/s11356-019-04153-x.

3. Cao, Z.; Jung, D.; Olszewski, M.P.; Arauzo, P.J.; Kruse, A. Hydrothermal carbonization of biogas digestate: Effect of digestate origin and process conditions. *Waste Manag.* **2019**, *100*, 138–150, doi:10.1016/j.wasman.2019.09.009.

4. Opatokun, S.A.; Yousef, L.F.; Strezov, V. Agronomic assessment of pyrolysed food waste digestate for sandy soil management. *J. Environ. Manage.* **2017**, *187*, 24–30, doi:10.1016/j.jenvman.2016.11.030.

5. Zhao, X.; Becker, G.C.; Faweya, N.; Rodriguez Correa, C.; Yang, S.; Xie, X.; Kruse, A. Fertilizer and activated carbon production by hydrothermal carbonization of digestate. *Biomass Convers. Biorefinery* **2018**, *8*, 423–436, doi:10.1007/s13399-017-0291-5.

6. Ambaye, T.G.; Rene, E.R.; Dupont, C.; Wongrod, S.; van Hullebusch, E.D. Anaerobic Digestion of Fruit Waste Mixed With Sewage Sludge Digestate Biochar: Influence on Biomethane Production. *Front. Energy Res.* **2020**, *8*, doi:10.3389/fenrg.2020.00031.

7. Pawlak-Kruczek, H.; Niedzwiecki, L.; Sieradzka, M.; Mlonka-Mędrala, A.; Baranowski, M.; Serafin-Tkaczuk, M.; Magdziarz, A. Hydrothermal carbonization of agricultural and municipal solid waste digestates – Structure and energetic properties of the solid products. *Fuel* **2020**, *275*, 117837, doi:10.1016/j.fuel.2020.117837.

8. Pawlak-Kruczek, H.; Urbanowska, A.; Yang, W.; Brem, G.; Magdziarz, A.; Seruga, P.; Niedzwiecki, L.; Pozarlik, A.; Mlonka-Mędrala, A.; Kabsch-Korbutowicz, M.; et al. Industrial Process Description for the Recovery of Agricultural Water From Digestate. *J. Energy Resour. Technol.* **2020**, *142*, doi:10.1115/1.4046141.

9. Urbanowska, A.; Kabsch-Korbutowicz, M.; Wnukowski, M.; Seruga, P.; Baranowski, M.; Pawlak-Kruczek, H.; Serafin-Tkaczuk, M.; Krochmalny, K.; Niedzwiecki, L. Treatment of Liquid By-Products of Hydrothermal Carbonization (HTC) of Agricultural Digestate Using Membrane Separation. *Energies* **2020**, *13*, 262, doi:10.3390/en13010262.

10. Calamai, A.; Chiaramonti, D.; Casini, D.; Masoni, A.; Palchetti, E. Short-Term Effects of Organic Amendments on Soil Properties and Maize (Zea maize L.) Growth. *Agriculture* **2020**, *10*, 158, doi:10.3390/agriculture10050158.

11. Bekiaris, G.; Peltre, C.; Jensen, L.S.; Bruun, S. Using FTIR-photoacoustic spectroscopy for phosphorus speciation analysis of biochars. *Spectrochim. Acta Part A Mol. Biomol. Spectrosc.* **2016**, *168*, 29–36, doi:10.1016/j.saa.2016.05.049.

12. Monlau, F.; Francavilla, M.; Sambusiti, C.; Antoniou, N.; Solhy, A.; Libutti, A.; Zabaniotou, A.; Barakat, A.; Monteleone, M. Toward a functional integration of anaerobic digestion and pyrolysis for a sustainable resource management. Comparison between solid-digestate and its derived pyrochar as soil amendment. *Appl. Energy* **2016**, *169*, 652–662, doi:10.1016/j.apenergy.2016.02.084.

13. Zhang, B.; Joseph, G.; Wang, L.; Li, X.; Shahbazi, A. Thermophilic anaerobic digestion of cattail and hydrothermal carbonization of the digestate for co-production of biomethane and hydrochar. *J. Environ. Sci. Heal. Part A* **2020**, *55*, 230–238, doi:10.1080/10934529.2019.1682367.

14. Zhang, T.; He, X.; Deng, Y.; Tsang, D.C.W.; Jiang, R.; Becker, G.C.; Kruse, A. Phosphorus recovered from digestate by hydrothermal processes with struvite crystallization and its potential as a fertilizer. *Sci. Total Environ.* **2020**, *698*, 134240, doi:10.1016/j.scitotenv.2019.134240.

15. Zhang, T.; Wu, X.; Shaheen, S.M.; Zhao, Q.; Liu, X.; Rinklebe, J.; Ren, H. Ammonium nitrogen recovery from digestate by hydrothermal pretreatment followed by activated hydrochar sorption. *Chem. Eng. J.* **2020**, *379*, 122254, doi:10.1016/j.cej.2019.122254.

16. Piccoli, I.; Torreggiani, A.; Pituello, C.; Pisi, A.; Morari, F.; Francioso, O. Automated image analysis and hyperspectral imagery with enhanced dark field microscopy applied to biochars produced at different temperatures. *Waste Manag.* **2020**, *105*, 457–466, doi:10.1016/j.wasman.2020.02.037.

17. Calamai, A.; Palchetti, E.; Masoni, A.; Marini, L.; Chiaramonti, D.; Dibari, C.; Brilli, L. The Influence of Biochar and Solid Digestate on Rose-Scented Geranium (Pelargonium graveolens L'Hér.) Productivity and Essential Oil Quality. *Agronomy* **2019**, *9*, 260, doi:10.3390/agronomy9050260.

18. Zhang, D.; Zhang, J.; Xia, C. Multi-Complementary Model for Long-Term Tracking. *Sensors* **2018**, *18*, 527, doi:10.3390/s18020527.

19. Zhang, J.-J.; Fan, H.-X.; Dai, X.-H.; Yuan, S.-J. Digested sludge-derived three-dimensional hierarchical porous carbon for high-performance supercapacitor electrode. *R. Soc. Open Sci.* **2018**, *5*, 172456, doi:10.1098/rsos.172456.

20. Chang, S.; Zhang, Z.; Cao, L.; Ma, L.; You, S.; Li, W. Co-gasification of digestate and lignite in a downdraft fixed bed gasifier: Effect of temperature. *Energy Convers. Manag.* **2020**, *213*, 112798, doi:10.1016/j.enconman.2020.112798.

21. Pecchi, M.; Patuzzi, F.; Benedetti, V.; Di Maggio, R.; Baratieri, M. Thermodynamics of hydrothermal carbonization: Assessment of the heat release profile and process enthalpy change. *Fuel Process. Technol.* **2020**, *197*, 106206, doi:10.1016/j.fuproc.2019.106206.

22. Rodriguez Alberto, D.; Stojak Repa, K.; Hegde, S.; Miller, C.W.; Trabold, T.A. Novel Production of Magnetite Particles via Thermochemical Processing of Digestate From Manure and Food Waste. *IEEE Magn. Lett.* **2019**, *10*, 1–5, doi:10.1109/LMAG.2019.2931975.

23. Opatokun, S.A.; Kan, T.; Al Shoaibi, A.; Srinivasakannan, C.; Strezov, V. Characterization of Food Waste and Its Digestate as Feedstock for Thermochemical Processing. *Energy & Fuels* **2016**, *30*, 1589–1597, doi:10.1021/acs.energyfuels.5b02183.

24. Luo, Z.; Wang, D.; Zeng, W.; Yang, J. Removal of refractory organics from piggery bio-treatment effluent by the catalytic ozonation process with piggery biogas residue biochar as the catalyst. *Sci. Total Environ.* **2020**, *734*, 139448, doi:10.1016/j.scitotenv.2020.139448.

25. Miliotti, E.; Casini, D.; Rosi, L.; Lotti, G.; Rizzo, A.M.; Chiaramonti, D. Lab-scale pyrolysis and hydrothermal carbonization of biomass digestate: Characterization of solid products and compliance with biochar standards. *Biomass and Bioenergy* **2020**, *139*, 105593, doi:10.1016/j.biombioe.2020.105593.

26. Cao, Z.; Hülsemann, B.; Wüst, D.; Illi, L.; Oechsner, H.; Kruse, A. Valorization of maize silage digestate from two-stage anaerobic digestion by hydrothermal carbonization. *Energy Convers. Manag.* **2020**, *222*, 113218, doi:10.1016/j.enconman.2020.113218.

27. Nisticò, R.; Guerretta, F.; Benzi, P.; Magnacca, G.; Mainero, D.; Montoneri, E. Thermal Conversion of Municipal Biowaste Anaerobic Digestate to Valuable Char. *Resources* **2019**, *8*, 24, doi:10.3390/resources8010024.

28. Tsai, W.-T.; Fang, Y.-Y.; Cheng, P.-H.; Lin, Y.-Q. Characterization of mesoporous biochar produced from biogas digestate implemented in an anaerobic process of large-scale hog farm. *Biomass Convers. Biorefinery* **2018**, *8*, 945–951, doi:10.1007/s13399-018-0344-4.

29. Antoniou, N.; Monlau, F.; Sambusiti, C.; Ficara, E.; Barakat, A.; Zabaniotou, A. Contribution to Circular Economy options of mixed agricultural wastes management: Coupling anaerobic digestion with gasification for enhanced energy and material recovery. *J. Clean. Prod.* **2019**, *209*, 505–514, doi:10.1016/j.jclepro.2018.10.055.

30. Barbanera, M.; Pelosi, C.; Taddei, A.R.; Cotana, F. Optimization of bio-oil production from solid digestate by microwave-assisted liquefaction. *Energy Convers. Manag.* **2018**, *171*, 1263–1272, doi:10.1016/j.enconman.2018.06.066.

31. Aragón-Briceño, C.; Ross, A.B.; Camargo-Valero, M.A. Evaluation and comparison of product yields and bio-methane potential in sewage digestate following hydrothermal treatment. *Appl. Energy* **2017**, *208*, 1357–1369, doi:10.1016/j.apenergy.2017.09.019.

32. Weidemann, E.; Buss, W.; Edo, M.; Mašek, O.; Jansson, S. Influence of pyrolysis temperature and production unit on formation of selected PAHs, oxy-PAHs, N-PACs, PCDDs, and PCDFs in biochar—a screening study. *Environ. Sci. Pollut. Res.* **2018**, *25*, 3933–3940, doi:10.1007/s11356-017-0612-z.

33. Marin-Batista, J.D.; Mohedano, A.F.; Rodríguez, J.J.; de la Rubia, M.A. Energy and phosphorous recovery through hydrothermal carbonization of digested sewage sludge. *Waste Manag.* **2020**, *105*, 566–574, doi:10.1016/j.wasman.2020.03.004.

34. Parmar, K.R.; Ross, A.B. Integration of Hydrothermal Carbonisation with Anaerobic Digestion; Opportunities for

Valorisation of Digestate. *Energies* **2019**, *12*, 1586, doi:10.3390/en12091586.

35. Luo, C.; Lü, F.; Shao, L.; He, P. Application of eco-compatible biochar in anaerobic digestion to relieve acid stress and promote the selective colonization of functional microbes. *Water Res.* **2015**, *68*, 710–718, doi:10.1016/j.watres.2014.10.052.

36. Li, Q.; Xu, M.; Wang, G.; Chen, R.; Qiao, W.; Wang, X. Biochar assisted thermophilic co-digestion of food waste and waste activated sludge under high feedstock to seed sludge ratio in batch experiment. *Bioresour. Technol.* **2018**, *249*, 1009–1016, doi:10.1016/j.biortech.2017.11.002.

37. Lü, F.; Luo, C.; Shao, L.; He, P. Biochar alleviates combined stress of ammonium and acids by firstly enriching Methanosaeta and then Methanosarcina. *Water Res.* **2016**, *90*, 34–43, doi:10.1016/j.watres.2015.12.029.

38. Jang, H.M.; Choi, Y.-K.; Kan, E. Effects of dairy manure-derived biochar on psychrophilic, mesophilic and thermophilic anaerobic digestions of dairy manure. *Bioresour. Technol.* **2018**, *250*, 927–931, doi:10.1016/j.biortech.2017.11.074.

39. Qin, Y.; Wang, H.; Li, X.; Cheng, J.J.; Wu, W. Improving methane yield from organic fraction of municipal solid waste (OFMSW) with magnetic rice-straw biochar. *Bioresour. Technol.* **2017**, *245*, 1058–1066, doi:10.1016/j.biortech.2017.09.047.

40. Cai, J.; He, P.; Wang, Y.; Shao, L.; Lü, F. Effects and optimization of the use of biochar in anaerobic digestion of food wastes. *Waste Manag. Res. J. a Sustain. Circ. Econ.* **2016**, *34*, 409–416, doi:10.1177/0734242X16634196.

41. Fagbohungbe, M.O.; Herbert, B.M.J.; Hurst, L.; Li, H.; Usmani, S.Q.; Semple, K.T. Impact of biochar on the anaerobic digestion of citrus peel waste. *Bioresour. Technol.* **2016**, *216*, 142–149, doi:10.1016/j.biortech.2016.04.106.

42. Shanmugam, S.R.; Adhikari, S.; Nam, H.; Kar Sajib, S. Effect of bio-char on methane generation from glucose and aqueous phase of algae liquefaction using mixed anaerobic cultures. *Biomass and Bioenergy* **2018**, *108*, 479–486, doi:10.1016/j.biombioe.2017.10.034.

43. Shen, Y.; Linville, J.L.; Ignacio-de Leon, P.A.A.; Schoene, R.P.; Urgun-Demirtas, M. Towards a sustainable paradigm of waste-to-energy process: Enhanced anaerobic digestion of sludge with woody biochar. *J. Clean. Prod.* **2016**, *135*, 1054–1064, doi:10.1016/j.jclepro.2016.06.144.

44. Sunyoto, N.M.S.; Zhu, M.; Zhang, Z.; Zhang, D. Effect of biochar addition on hydrogen and methane production in two-phase anaerobic digestion of aqueous carbohydrates food waste. *Bioresour. Technol.* **2016**, *219*, 29–36, doi:10.1016/j.biortech.2016.07.089.

45. Wang, G.; Li, Q.; Gao, X.; Wang, X.C. Synergetic promotion of syntrophic methane production from anaerobic digestion of complex organic wastes by biochar: Performance and associated mechanisms. *Bioresour. Technol.* **2018**, *250*, 812–820, doi:10.1016/j.biortech.2017.12.004.

46. Shen, Y.; Forrester, S.; Koval, J.; Urgun-Demirtas, M. Yearlong semi-continuous operation of thermophilic two-stage anaerobic digesters amended with biochar for enhanced biomethane production. *J. Clean. Prod.* **2017**, *167*, 863–874, doi:10.1016/j.jclepro.2017.05.135.

47. Wang, D.; Ai, J.; Shen, F.; Yang, G.; Zhang, Y.; Deng, S.; Zhang, J.; Zeng, Y.; Song, C. Improving anaerobic digestion of easy-acidification substrates by promoting buffering capacity using biochar derived from vermicompost. *Bioresour. Technol.* **2017**, *227*, 286–296, doi:10.1016/j.biortech.2016.12.060.

48. Shen, Y.; Linville, J.L.; Urgun-Demirtas, M.; Schoene, R.P.; Snyder, S.W. Producing pipeline-quality biomethane via anaerobic digestion of sludge amended with corn stover biochar with in-situ CO2 removal. *Appl. Energy* **2015**, *158*, 300–309, doi:10.1016/j.apenergy.2015.08.016.
